# European Association for Endoscopic Surgery (EAES) consensus on Indocyanine Green (ICG) fluorescence-guided surgery

**DOI:** 10.1007/s00464-023-09928-5

**Published:** 2023-02-13

**Authors:** E. Cassinotti, M. Al-Taher, S. A. Antoniou, A. Arezzo, L. Baldari, L. Boni, M. A. Bonino, N. D. Bouvy, R. Brodie, T. Carus, M. Chand, M. Diana, M. M. M. Eussen, N. Francis, A. Guida, P. Gontero, C. M. Haney, M. Jansen, Y. Mintz, S. Morales-Conde, B. P. Muller-Stich, K. Nakajima, F. Nickel, M. Oderda, P. Parise, R. Rosati, M. P. Schijven, G. Silecchia, A. S. Soares, S. Urakawa, N. Vettoretto

**Affiliations:** 1 Department of General and Minimally Invasive Surgery, Fondazione IRCCS Ca’ Granda Ospedale Maggiore Policlinico di Milano, University of Milan, Via Francesco Sforza 35, 20121 Milan, Italy; 2 Research Institute Against Digestive Cancer (IRCAD), Strasbourg, France; 3 Department of Surgery, Papageorgiou General Hospital, Thessaloniki, Greece; 4 Department of Surgical Sciences, University of Torino, Turin, Italy; 5 Department of Surgery, Geneva University Hospitals, Geneva, Switzerland; 6 Department of Surgery, Maastricht University Medical Center, Maastricht, The Netherlands; 7 Department of General Surgery, Hadassah-Hebrew University Medical Center, Jerusalem, Israel; 8 Niels-Stensen-Kliniken, Elisabeth-Hospital, Thuine, Germany; 9 Wellcome/EPSRC Centre for Interventional and Surgical Sciences (WEISS), University College London, London, UK; 10 Division of Surgery and Interventional Sciences, University College London, London, UK; 11 IHU Strasbourg, Institute of Image-Guided Surgery and IRCAD, Research Institute Against Cancer of the Digestive System, Strasbourg, France; 12 Department of General Surgery, Yeovil District Hospital NHS Foundation Trust, Yeovil, UK; 13 Department of Medico-Surgical Sciences and Translation Medicine, Faculty of Medicine and Psychology, Sapienza University of Rome, Rome, Italy; 14 Division of Urology, Department of Surgical Science, AOU Città della Salute e della Scienza di Torino, University of Turin, Turin, Italy; 15 Department of General, Visceral, and Transplantation Surgery, Heidelberg University Hospital, Heidelberg, Germany; 16 Department of Surgery, Amsterdam UMC, University of Amsterdam, Amsterdam, The Netherlands; 17 Faculty of Medicine, Hebrew University of Jerusalem, Jerusalem, Israel; 18 Unit of Innovation in Minimally Invasive Surgery, Department of General Surgery, University Hospital Virgen del Rocío, University of Sevilla, Seville, Spain; 19 Department of Gastroenterological Surgery, Osaka University Graduate School of Medicine, Osaka, Japan; 20 U.O.C. Chirurgia Generale, Policlinico di Abano Terme, Abano Terme, PD Italy; 21 Department of Gastrointestinal Surgery, San Raffaele Hospital, Milan, Italy; 22 Department of Surgery, Amsterdam UMC, University of Amsterdam, Amsterdam, North Holland The Netherlands; 23 Amsterdam Gastroenterology and Metabolism, Amsterdam UMC, Amsterdam, North Holland The Netherlands; 24 Amsterdam Public Health, Digital Health, Amsterdam UMC, Amsterdam, North Holland The Netherlands; 25 U.O.C. Chirurgia Generale, ASST Spedali Civili di Brescia P.O. Montichiari, Ospedale di Montichiari, Montichiari, Italy

**Keywords:** Indocyanine green, ICG, Fluorescence-guided surgery, Laparoscopic surgery, Consensus, Statement recommendation

## Abstract

**Background:**

In recent years, the use of Indocyanine Green (ICG) fluorescence-guided surgery during open and laparoscopic procedures has exponentially expanded across various clinical settings. The European Association of Endoscopic Surgery (EAES) initiated a consensus development conference on this topic with the aim of creating evidence-based statements and recommendations for the surgical community.

**Methods:**

An expert panel of surgeons has been selected and invited to participate to this project. Systematic reviews of the PubMed, Embase and Cochrane libraries were performed to identify evidence on potential benefits of ICG fluorescence-guided surgery on clinical practice and patient outcomes. Statements and recommendations were prepared and unanimously agreed by the panel; they were then submitted to all EAES members through a two-rounds online survey and results presented at the EAES annual congress, Barcelona, November 2021.

**Results:**

A total of 18,273 abstracts were screened with 117 articles included. 22 statements and 16 recommendations were generated and approved. In some areas, such as the use of ICG fluorescence-guided surgery during laparoscopic cholecystectomy, the perfusion assessment in colorectal surgery and the search for the sentinel lymph nodes in gynaecological malignancies, the large number of evidences in literature has allowed us to strongly recommend the use of ICG for a better anatomical definition and a reduction in post-operative complications.

**Conclusions:**

Overall, from the systematic literature review performed by the experts panel and the survey extended to all EAES members, ICG fluorescence-guided surgery could be considered a safe and effective technology. Future robust clinical research is required to specifically validate multiple organ-specific applications and the potential benefits of this technique on clinical outcomes.

**Supplementary Information:**

The online version contains supplementary material available at 10.1007/s00464-023-09928-5.

In the last few years, with the growth and progressive spread of minimally invasive surgical techniques, several tools and instruments have been developed to enhance surgeons’ performance and patient safety and potentially decrease the risk of human errors [1]. Among these tools, such as high-definition visual systems like 4 K or 3D imaging, is Indocyanine Green (ICG) fluorescence-guided surgery (FGS), which is a modality of intraoperative imaging system that could significantly contribute to intraoperative anatomical navigation and improve decision-making during the surgical procedure [2, 3].

FGS is based on the ability of a dye (ICG) to emit a fluorescent signal when excited with a light source at a specific wavelength (near-infrared light spectrum of 700–900 nm). For several decades, clinical use of ICG has been reported in the assessment of hepatic blood flow, the assessment of choroidal blood flow and the measurement of cardiac output. ICG is rapidly and exclusively excreted into the bile. Due to its well-established clinical applications, relatively low cost, and extremely low toxic dose/reported allergic reactions, ICG is currently the most employed fluorophore in general surgery clinical settings [4–6]. As regards technology development, multiple near-infrared visual systems have already been developed, either using a laser beam or LED light sources, both for laparoscopic and robotic surgery, and it is to be expected that more systems will be developed and introduced in the near future. Furthermore, ICG fluorescence imaging has also been demonstrated to have a short learning curve, it does not require complex equipment in the operating room, and it is not time-consuming without interfering with the surgical workflow [7].

Since ICG fluorescence imaging is one of the most promising and rapidly developing technical innovations in surgery of the last decade, the clinical applications of this technology have expanded exponentially [4], including fluorescence cholangiography in laparoscopic cholecystectomy [8, 9], lymph node identification and mapping in oncologic surgery [10, 11] and bowel anastomotic perfusion assessment [12, 13].

In the last years, the number of studies published regarding ICG-FGS have rapidly grown, suggesting that this technology is safe, feasible and could represent a benefit for both surgeons by simplifying and guiding some procedures, and for patients, in terms of reducing post-operative complications. Nevertheless, there is still significant variability in clinical use and technical details of use, such as dose, concentration and timing of ICG administration. Additionally, there are issues regarding whether or not fluorescence-guided surgery could potentially be considered the standard of care in some surgical applications [14].

Therefore, the European Association of Endoscopic Surgery (EAES) sponsored this consensus development conference on the use of ICG Fluorescence-guided Surgery to critically review all available data on fluorescence imaging in abdominal surgery. The aim of this project was to provide consensus statements and to develop recommendations for the surgical community based on the available evidence and inputs of some of the most experienced European experts and opinion makers in this field.

## Materials and methods

The objective of this Consensus on the use of ICG fluorescence-guided surgery was to provide evidence-based recommendations on the use of vision enhanced by ICG fluorescence compared with standard vision in different clinical settings. The scope of this project consisted of three main parts: (i) general topics, (ii) organ-specific data and (iii) ongoing trials. Within each of these topics, subcategories have been defined.

‘General topics’ included (a) cognitive load, (b) costs and cost-effectiveness.

‘Organ specific’ topics included (a) cholecystectomy, (b) perfusion assessment in colorectal surgery, (c) lymphatic mapping in colorectal surgery, (d) bariatric surgery, (e) spleen and adrenal surgery, (f) pancreatic surgery, (g) liver surgery, (h) perfusion assessment in Upper GI surgery, (i) lymphatic mapping in Upper GI surgery, (j) urology and (k) gynaecology.

### Research team and search strategy

An expert panel of surgeons functioned as the coordinating team (EC, AA, LB and NV); they formulated a list of questions related to each topic to be specifically addressed, which guided the literature research (Table 1).

**Table 1:** List of questions regarding the use of ICG-guided fluorescence surgery to be addressed

*General topics*		
1		Does intraoperative ICG fluorescence introduce a higher cognitive load for the surgeon compared to standard laparoscopic systems?
2		What is the impact of intraoperative ICG fluorescence on costs?
*Organ-specific topics*		
1		Should ICG fluorescence versus standard surgery be used in laparoscopic cholecystectomy?
	1.1	Does intraoperative ICG fluorescence provide better visualisation of biliary anatomy in laparoscopic cholecystectomy compared to standard light visualisation?
	1.2	Does intraoperative ICG fluorescence reduce post-operative complications in laparoscopic cholecystectomy compared to standard light visualisation?
2		Should ICG fluorescence versus standard surgery be used in colorectal cancer surgery?
	2.1	Does intraoperative ICG fluorescence angiography reduce morbidities in colorectal cancer surgery?
	2.2	Should ICG fluorescence versus standard surgery be used for lymphadenectomy in colorectal cancer surgery?
3		Should ICG fluorescence versus standard surgery be used in bariatric surgery?
4		Should ICG fluorescence versus standard surgery be used in abdominal endocrine surgery?
5		Should ICG fluorescence versus standard surgery be used in pancreatic surgery?
6		Should ICG fluorescence versus standard surgery be used in liver surgery?
	6.1	Does intraoperative ICG fluorescence reduce bile leaks in liver surgery?
	6.2	Does intraoperative ICG fluorescence help in tumour identification and resection in liver surgery?
7		Should ICG fluorescence versus standard surgery be used in UpperGI cancer surgery?
	7.1	Should ICG fluorescence versus standard surgery be used for lymphadenectomy in UpperGI cancer surgery?
	7.2	Does intraoperative ICG fluorescence angiography reduce anastomotic leak in UpperGI cancer surgery
8		Should ICG fluorescence versus standard surgery be used in urologic surgery?
9		Should ICG fluorescence versus standard surgery be used in gynecologic surgery?

The coordinators invited 12 expert surgeons, members of the EAES research and technology committee with recognised expertise on the topic to join the panel of experts. Each was asked to nominate at least one young surgical researcher to participate. An international research team consisted of 12 young surgical researchers was formed to review and evaluate the existing literature on the use of ICG fluorescence-guided surgery. Each young researcher was mentored by an expert surgeon. The final list of topics was approved by the experts and subsequently divided among the teams.

All searches were performed in PubMed, Embase and Cochrane electronic libraries starting from September 2019 with no limitation regarding the year of publication or language. Due to the pandemic restrictions, the difficulty of the research group to meet in person, the literature search was extended and updated until November 2020. The composition of search strings has been discussed and approved by a librarian from the University of Milan. Search strings are provided in Supplementary Materials.

Study inclusion criteria were: Randomised Controlled Trials (RCTs), prospective and retrospective observational comparative studies. Case reports and non-comparative studies were excluded as well as studies in children under 12 years of age and papers not in the English language.

All search hits were screened by topic and reviewed by two team members for eligibility, based on title and abstract. If considered eligible, full-text articles were reviewed and summarised. In cases of disagreement, the coordinators acted as referees and made the final decision.

### Data extraction and appraisal of the methodological quality of the studies

Standardised data extraction forms were used across all topics. A uniform Excel database template for entering the data extracted from the selected papers was provided to all participants. Important outcome measures with respect to ICG fluorescence-guided surgery compared to standard light imaging were included in the template, such as operating time, conversion to open surgery, hospital stay, post-operative pain, adverse events, post-operative complications and mortality. The participants were encouraged to add any other outcome measure if necessary. The template contained predefined fields for noting important information for each study, like population characteristics, detailed information about the surgical procedure, the experience level of the surgeons, etc., and the results for each outcome, including the effect, size and statistical significance, where appropriate.

A PRISMA chart was completed for each literature search according to recommendations [15]. The methodological quality of included RCT was assessed using the Cochrane risk of bias score [16].

After data extraction, the teams worked out a presentation on their topic and a more comprehensive summary of their findings, including a flowchart of the selection of articles, a description of the population, a summary of the papers, conclusions, statements and recommendations.

If the number and quality of the studies included in the final analysis were considerate appropriate, metanalysis was conducted to answer PICO questions and prepare the related statements.

With each “Statement”, the level of evidence was given. The original Centre for Evidence-Based Medicine levels of evidence (LoE) system was used [17], which defines five levels, ranging from Level 1 (highest evidence) to Level 5 (lowest evidence). This tool allows for grading levels down on the basis of study quality, imprecision, inconsistency between studies, etc. It also allows to grade up in case of large or very large effect size.

With each ‘Recommendation’, the level of recommendation was given. These were graded as ‘strong’ or ‘weak’ or ‘no recommendation’ according to the GRADE system. GRADE is a systematic and explicit approach to judging the quality of evidence and strength of recommendations. GRADE specifically assesses methodological flaws, consistency of results across different studies, the generalizability of research results and treatment effectiveness. When data were considered sufficient, consensus statements were prepared by each team and scored with a grade of recommendation (GoR) [18–20].

### Consensus development process

A face-to-face first consensus meeting was held in Krakow on 20 January 2020 to present all findings and preliminary drafted consensus statements and recommendations, which were finalised during further virtual meetings. A modified Delphi method was used, as anonymity was not applicable in our situation [21, 22]. All statements and recommendations were shared with the proposed LoE and subjected to voting for agreement or disagreement. In the case of 100% consensus, the statements and recommendations were accepted. Where there was a lack of Consensus, the research team responsible for that topic presented the underlying evidence and rationale for their statement. After discussion, further voting rounds were conducted until an agreement was reached.

All finalised recommendations and statements with LoE and GoR were planned to be presented at a dedicated session during the 28th EAES congress in Krakow 2020 to be voted by EAES delegates. Unfortunately, due to the Covid pandemic, the 2020 EAES congress was cancelled. As mentioned, the working group then organised further online meetings and updated the literature search up to November 2020. Previous results were revised based on updated literature. In April and June 2021 online survey among all EAES members was organised and consisted of two-rounds of voting, until an agreement greater than 75% on each recommendation was obtained.

An online repository was created in order to obtain access to the full Consensus protocol, literature search strategies, PRISMA flow charts, and full text of articles included in the final analysis for each topic. PRISMA charts are provided in Supplementary Materials.

225 EAES members participated in the survey and voted for each recommendation: (a) Agree with the above-mentioned recommendation (b) Disagree or (c) Don’t know/No opinion.

For each topic analyzed, in addition to the statements and recommendations, a brief discussion on the results obtained has been reported.

## Results

The literature searches yielded 18,273 abstracts to be screened. In total, 117 articles were included and reviewed in detail to define 22 consensus statements and 16 recommendations. 227 EAES members completed first round online survey; a second-round survey, completed by 193 EAES members, was carried out only for recommendations that did not reach 75% agreement.

### General topics

#### Cognitive load

The systematic literature search retrieved a total of 210 articles, which were independently screened by two experts (MJ and MS), with 10 cases of disagreement on inclusion, which were resolved during a discussion in the presence of a third independent collaborator. No articles were found eligible for inclusion.

Cognitive load and how it may be affected has not been studied when using fluorescence in laparoscopy to date. Studies about the use of fluorescence in laparoscopy report mostly validity and reliability and sometimes state that surgeons found fluorescence “easy to use”.

No studies reported experienced workload or used questionnaires like the NASA TLX to measure experienced cognitive load.

*Recommendation*

With the currently available evidence, no recommendations regarding cognitive load can be provided.

#### Cost-effectiveness

Due to the lack of articles specifically focussing on cost-effectiveness of Indocyanine Green fluorescence-guided surgery, no statements and recommendations were included in the EAES members survey for this topic.

Nevertheless, all members of the expert panel had recently participated and published a Health Technology Assessment (HTA) in order to investigate the impact of fluorescence surgery on costs and cost-effectiveness. In April 2020, Vettoretto et al., in cooperation with SICE (Italian Society of Endoscopic Surgery), designed a study where an HTA approach was implemented to investigate the economic, social, ethical, and organisational implications related to the adoption of ICG fluorescence-guided surgery compared to standard vision surgery [23].

With the support of a multidisciplinary team, qualitative and quantitative data were collected by means of literature evidence, validated questionnaires and self-reported interviews, considering the dimensions resulting from the EUnetHTA Core Model.

The multidisciplinary team included expert surgeons, healthcare economists, managerial engineers, HTA and methodology experts, statisticians and clinical engineers.

Systematic reviews were conducted to detect evidence in the literature concerning the use of ICG fluorescence-guided surgery in several clinical settings. To assess the costs of this technology, an activity-based costing analysis (ABC) was implemented to measure, record, and calculate both the cost and the performance of activities [24]. In colorectal, particularly in rectal surgery, stronger evidence (confirmed by the experts’ opinion and by real-world practice) supports a benefit in the use of ICG-FGS, with a significant reduction of complications, which could be translated into advantages in the length of hospitalisation. The final evaluation may depend on overcoming the phase of technological introduction, thus defining potential advantages and greater practicality of use.

The economic evaluation of the patient’s pathway was then integrated with cost-effectiveness and budget impact analyses. The cost-effectiveness evaluation was developed to define the technology presenting a better trade-off between the efficacy achieved and the costs absorbed. A budget impact analysis (BIA) was conducted to estimate the financial effects of both the use and the consequent spread of new healthcare technology in a setting with limited resources [25]. The budget impact analysis predicted a reduction in costs, thus freeing up some economic and organisational resources. From an economic point of view, results suggested the opportunity to achieve significant economic savings, ranging from 4 to 8%, even in a conservative scenario of analysis, showing that investments in this field could be feasible and sustainable.

Also, multiple HTA dimensions (safety, efficacy, equity etc.) were evaluated through specific qualitative questionnaires to gather clinicians’ perceptions regarding their ICG fluorescence-guided surgery use. Results showed that ICG could be the preferable solution from an effectiveness point of view (average value: 0.54 vs 2.14, *p*-value = 0.000). ICG Fluorescence would thus be favourable to patients’ reported outcomes, the detection rate, image quality, the visualisation of perfusion, the precision of the surgical technique, and the separation/discrimination between healthy and not healthy tissues. The use of ICG is perceived as improving the precision of the surgical technique, the identification of the blood vessels and the lymph node detection rate, allowing for better image quality compared with standard white light. Despite no static differences that emerged with regard to the safety aspect perceptions (average value: 0.81 vs 0.98, *p*-value > 0.05), ICG is related to a lower occurrence of surgical complications.

We refer to the consultation of the original study for further details on all aspects of this multidimensional HTA report [23].

### Organ-specific topics

#### Cholecystectomy

Over the last several years, the use of ICG fluorescence-guided surgery during laparoscopic cholecystectomy has emerged as a new technology allowing real-time enhanced visualisation and identification of extra-hepatic biliary structures without the use of radiation. As a result, proper identification of vital structures and high-risk areas that should be observed until dissection enables the key landmarks to be localised, is facilitated. One advantage of performing fluorescent cholangiography (FC) routinely is the ability to recognise the common bile duct before dissection; this has proven to be useful not only in the normal course of the procedure but also serves as a precautionary measure in the presence of anatomical variations or in certain conditions (e. g., the presence of inflamed tissue/acute cholecystitis settings) posing an increased risk for iatrogenic injury [26].

A total of 936 records were identified. Following screening for eligibility, 37 articles (representing unique studies) were included for potential data extraction and assessment of the risk of bias. However, 28 studies were further excluded due to inadequate study design or incompletely reported outcome data. Nine studies containing 2763 patients met inclusion criteria: two RCTs, four prospective studies and three large retrospective studies conducted on a prospectively maintained database [27–35]. One of the RCTs was a single-centre non-inferiority trial comparing FC with standard x-ray intraoperative cholangiogram (IOC), while the other RCT was a multicentre trial on 639 patients comparing the efficacy of fluorescence-guided surgery with the use of conventional white light vision. Also, other prospective or retrospective case-match studies used white light as the comparator. Given the low incidence reported in the literature of bile duct injuries during laparoscopic cholecystectomy (0,5–2%) [36], it is not possible to design a trial that uses this endpoint as the main outcome since, in order to demonstrate a possible statistical advantage in the use of FC, more than 4000 patients should be enrolled. Both RCTs used the rate of biliary structures visualisation as the primary endpoint: Dip et al. demonstrated that FC was statistically superior to white light in visualising extra-hepatic biliary ducts before surgical dissection of the Calot’s triangle; results were confirmed in the subgroup analysis of patients with BMI > 30 and surgery for acute cholecystitis; in the other RCT Lehrskov et al. showed that FC is not inferior to standard IOC in visualising critical junction between the cystic duct, common hepatic duct and common bile duct [33, 35].

The other studies analysed focussed on operative time, rate of conversion to open surgery and overall post-operative complications; no significant differences between the two techniques were reported in terms of complications, although they all showed that FC is a non-invasive adjunct to laparoscopic cholecystectomy, leading to improved patient outcomes with respect to operative times, decreased conversion to open procedures, and shorter length of hospitalisation [27–32, 34].

*Statements*

i. Fluorescent cholangiography during laparoscopic cholecystectomy improves the identification of the extra-hepatic biliary anatomy before and after dissection of Calot’s triangle, compared with standard intraoperative imaging (LoE: high).
ii. Fluorescent cholangiography during laparoscopic cholecystectomy may reduce operative time and conversion rate compared with standard intraoperative imaging (LoE: moderate/low)
iii. Fluorescent cholangiography during laparoscopic cholecystectomy in obese patients may improve identification of the extra-hepatic biliary anatomy before and after dissection of Calot’s triangle, compared with standard intraoperative imaging (LoE: low)
iv. Fluorescent cholangiography during laparoscopic cholecystectomy in case of acute cholecystitis may improve identification of the extra-hepatic biliary anatomy before and after dissection of Calot’s triangle, compared with standard intraoperative imaging (LoE: low)

*Recommendation*

We recommend the use of fluorescent cholangiography during laparoscopic cholecystectomy, whenever available, in order to improve the visualisation of the biliary structures.

Grade of recommendation: Strong.

This recommendation received a 75% agreement on the first round of the online survey.

### ICG Fluorescence for perfusion assessment in colorectal surgery

Anastomotic leak is one of the most important complications following colorectal surgery. It is well established that one of the main reasons for an anatomic leak is insufficient tissue perfusion making the anastomosis not heal correctly. For this reason, in the last years, we have observed an increase in the number of centres using ICG to evaluate perfusion during colorectal anastomosis [37, 38].

Of 1612 papers screened in the systematic review in this field, we included in our qualitative analysis 54 trials, and from them, 30 were included in our quantitative analysis [39–68]. Twenty-five of them were retrospective studies, three prospective not RCTs and two RCTs. The risk of bias assessment did not highlight any significant bias, but for the definition of anastomotic leak.

**Fig. 1:**
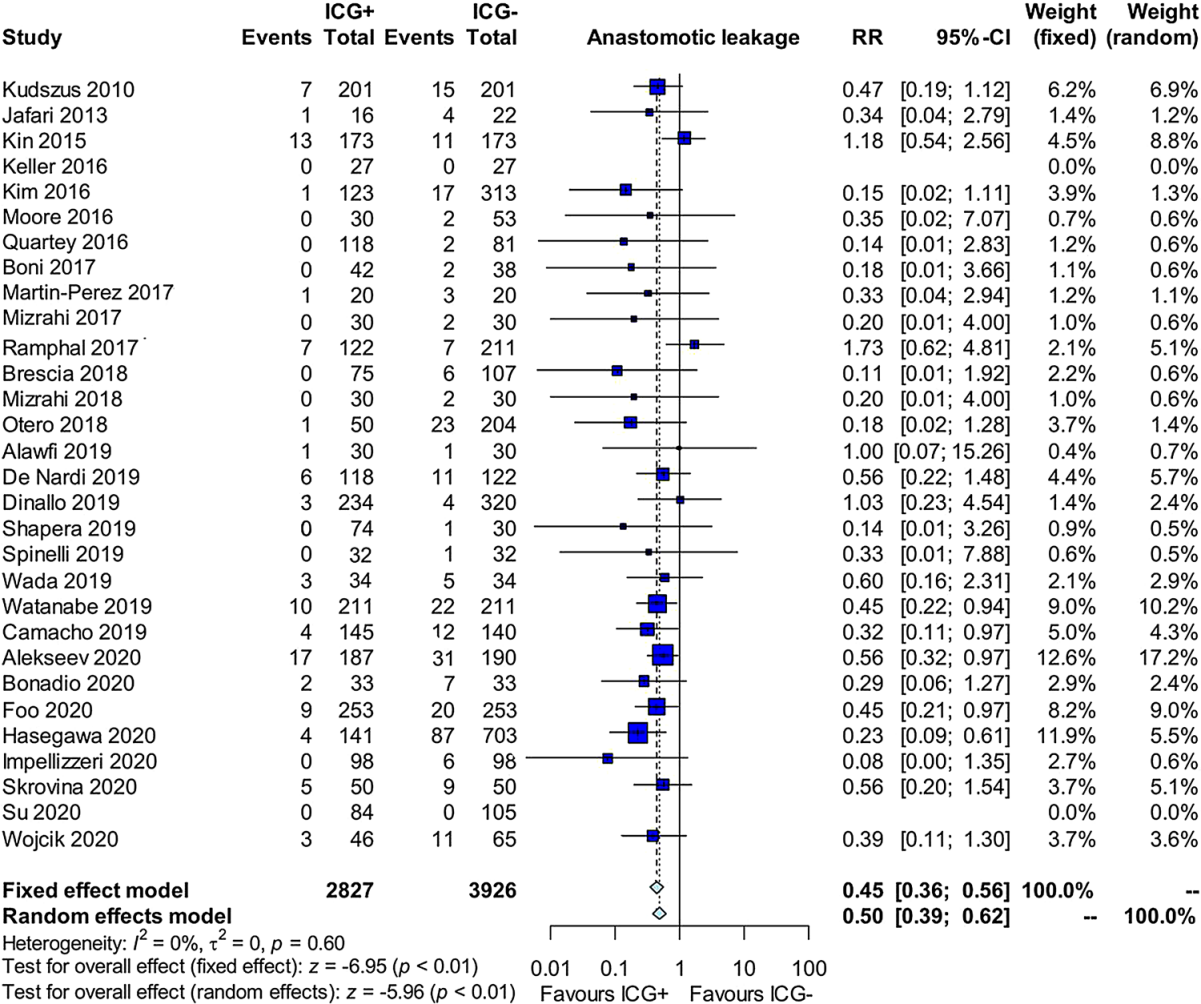
Forest plot of ICG Fluorescence-guided surgery versus ICG- on anastomotic leakage in colorectal surgery

The meta-analysis shows that the use of ICG is correlated with a reduction of events of anastomotic leakage, particularly in the rectum (RR = 0.32, IC 95% 0.22–0.49, *p* < 0.01, I2 = 0%) (Fig. 1). Moreover, the literature research described a change of anastomotic line after ICG injection in 10.3% of patients (10.2–12.5%). The meta-analysis shows a reduction in the overall post-operative complications (RR = 0.67, IC 95% 0.57–0.80, *p* < 0.01)). This is also true if we exclude from the list of complications the anastomotic leak (RR = 0.82, IC 95% 0.69–0.98, *p* = 0.03). Moreover, the use of ICG to assess perfusion during colorectal surgery reduces the post-operative length of hospital stay (MD − 0.67, IC 95%: − 1.06–− 0.27, *p* < 0.01). The operative time does not increase when using ICG (*p* = 0.37). A protective stoma was performed in only 44% of patients in the ICG group compared to 54% of the control group (*p* = 0.45).

Based on the literature research and meta-analytic results, we formulated the following statements and voted on the recommendations.

*Statements*

i. The use of ICG fluorescence to assess perfusion during colorectal surgery significantly reduces the risk of anastomotic leak (LoE: high).
ii. The use of ICG fluorescence in colorectal surgery can lead to a change in the resection line and/or refashioning the anastomosis (LoE: high).
iii. The use of ICG fluorescence to assess tissue perfusion while performing laparoscopic or robotic colorectal anastomosis does not affect the operative time (LoE: high).
iv. The use of ICG fluorescence to assess tissue perfusion in colorectal surgery reduces the length of hospital stay and overall morbidity (LoE: high).

*Recommendations*

The use of ICG fluorescence in colorectal surgery to assess tissue perfusion is recommended in order to reduce the risk of anastomotic leak.

Grade of recommendation: Strong.

The use of ICG fluorescence in colorectal surgery to assess tissue perfusion is suggested in order to reduce overall morbidity.

Grade of recommendation: Weak.

### ICG Fluorescence for lymphatic mapping in colorectal surgery

Among possible clinical applications of ICG fluorescence-guided surgery, is nodal navigation and real-time lymphography for cancers [69]. Following submucosal, subserosal or intradermal injection, ICG disperses in lymph, binds to lipoproteins, and is drained via lymphatic pathways and nodes. The resulting ICG fluorescent lymphography is a tool that could be used to guide the surgeon in performing a more precise lymphadenectomy and resection, and it may be a better option for overall patient outcomes.

In colorectal surgery, ICG fluorescent lymphography has been reported for assessing lymphatic routes both to evaluate the presence and value of sentinel nodes and, especially in laparoscopic right colectomy and flexure cancers, to highlight a watershed area around main vascular branches and facilitate more precise mesenteric dissection [70].

The literature search identified 388 abstracts. After screening, 38 papers were assessed for eligibility, although many were excluded being case reports or pilot studies on few patients. 12 studies were included in the qualitative data analysis [71–82]. No RCTs were found. All studies were prospective studies (most of which were pilot or feasibility studies on a small cohort of patients), mainly conducted from 2016 onwards. Two studies were performed exclusively on right colon and flexure cancers, while the others included various colonic resections. ICG tracer injection was performed endoscopically in the submucosal peritumoral area in 4 series, while the other 8 authors injected the dye at the beginning of laparoscopic abdominal exploration in the subserosal layer.

The primary outcome was mainly the feasibility of ICG fluorescent lymphangiography for lymphatic mapping in colon cancer. In all cases, ICG lymphography resulted safe and feasible. Different rates of sensitivity and accuracy (positive and negative predictive value) of the technique have been reported in the studies. Seven studies focussed on sentinel node retrieval, in some cases with combined intraoperative histopathological analysis of the nodes, while eight studies evaluated lymphatic flow in the mesenteric area. A single study was conducted with a case-match comparison with a historical cohort of patients aiming to compare the overall number of lymph nodes removed with and without ICG fluorescence guidance: ICG lymphography has resulted in a higher number of nodes retrieved.

Particularly for this research topic, studies are very heterogeneous, both for surgical technique and outcomes analysed, and hardly comparable. Future studies are mandatory to optimise ICG fluorescence-guided lymphography in the colorectal cancer setting.

*Statement*

ICG Fluorescent lymphatic mapping is safe and feasible to allow the identification of lymphatic anatomy during colectomy for cancer, although the clinical value is yet to be defined (LoE: low).

*Recommendation*

We recommend further research to standardise the technique of fluorescent lymphatic mapping during colorectal surgery and to investigate its clinical benefit.

Grade of recommendation: Strong.

This recommendation received a 92% agreement in the first round of the online survey.

### Bariatric surgery

The literature search identified 169 abstracts. Among 25 full texts assessed for eligibility, no RCT or prospective studies were found, and no studies could be included in the final analysis.

Regarding potential applications of ICG fluorescence-guided surgery in bariatrics, we report four retrospective studies conducted on a small cohort of patients; three of them dealt with the use of ICG fluorescence angiography to assess visceral perfusion in sleeve gastrectomy [83–85] and one pilot study reported experience with intra-operative leak test using a blend of methylene blue and indocyanine green during robotic gastric bypass surgery [86].

Hence, *due to insufficient evidence, no statement could be made about ICG fluorescence in bariatric surgery*.

*Recommendation*

We recommend further research on the use of ICG fluorescence in bariatric surgery to assess its potential clinical benefits.

Grade of recommendation: Strong.

This recommendation received a 75% agreement on the first round of the online survey.

### Spleen and adrenal surgery

The literature search identified 548 hits regarding spleen and adrenal surgery, although among them, 15 full texts were screened, and only 3 articles (case-series study and case reports) were assessed for eligibility. No RCT or prospective studies were found, and no studies could be included in the final analysis.

However, case reports and preliminary experiences showed multiple applications for implementation of ICG fluorescence imaging for surgery of the spleen and adrenal glands, such as clarification of spleen and adrenal vascular anatomy, margin identification in partial adrenalectomy and fluorescent angiography for spleen preservation in distal pancreatectomy [87–91].

Hence, *due to insufficient evidence, no statement could be made for ICG fluorescence in spleen and adrenal surgery*.

*Recommendation*

We recommend further research on the use of ICG fluorescence in spleen and adrenal surgery to assess its potential clinical benefits.

Grade of recommendation: Strong.

This recommendation received 77% agreement in the first round of the online survey.

### Pancreatic surgery

The literature search identified 1479 hits regarding pancreatic surgery, although among them, 24 full texts were screened, and only 1 article (retrospective study on 37 patients) was assessed for eligibility [92]. No RCT or prospective studies were found, and no studies could be included in the final analysis.

However, from our literature review, several case reports and preliminary experiences showed multiple potential applications of ICG fluorescence imaging to assist surgeons with real-time information in pancreatic surgery, such as tumour identification and tumour margin assessment, perfusion assessment of pancreatic and biliary anastomosis and identification of vascular anatomy [93–96].

Hence, *due to insufficient evidence, no statement could be made about ICG fluorescence in pancreatic surgery.*

*Recommendation*

We recommend further research on the use of ICG fluorescence in pancreatic surgery to assess its potential clinical benefits.

Grade of recommendation: Strong.

This recommendation received an 81% agreement in the first round of the online survey.

### Liver surgery

ICG fluorescence-guided surgery has gained popularity as intraoperative imaging modality in hepatobiliary surgery over the past decade, with a large number of studies conducted in Eastern countries, creating new interesting perspectives. Among multiple potential applications in this field, fluorescence imaging has proven to be helpful in identifying small subcapsular and superficial tumours but also to enhance deeper lesions identification and to obtain clear resection margins; it can also be used for visualizing extra-hepatic bile duct anatomy and hepatic segmental borders, increasing the accuracy and the easiness of open and minimally invasive hepatectomy especially for prevention of post-operative bile leaks [97, 98].

7536 abstracts were identified by literature search. Following elimination of duplicate records and elimination of articles meeting exclusion criteria, a total of 15 articles were included in final analysis, with only 1 RCT [99–113]. Eight studies compared conventional imaging (intraoperative ultrasound, IOUS) to ICG fluorescence imaging in identifying surface liver tumours. Overall ICG fluorescence was successfully able to identify superficial lesions, as small as 1–2 mm, that had previously not been identified preoperatively or with direct visualization. All studies agree that IOUS remains the gold standard, although some authors demonstrated that fluorescence imaging identified smaller lesions with higher accuracy than ultrasound and combining ICG with IOUS could significantly increase the sensitivity in locating superficial lesions. All authors also agree that ICG fluorescence for this application is highly reliable for tumors within 8–10 mm beneath the liver capsule, due to the limitations of infrared light to deeply penetrate into tissues.

As regards resection margins, nine studies analyzed the role of ICG fluorescence as a guidance during dissection: they showed that ICG can be especially beneficial in cases where liver tissue consistency is hardened secondary to other pathology, such as cirrhosis, making IOUS difficult and rendering tactile feedback unreliable; in fact, the lack of fluorescence in the normal tissue served as a guide for the dissection plane, allowing for higher R0 resection margin rates. Although two studies reported that ICG fluorescence technique might increase false positive rate of liver lesion detection due to the non-specific uptake of lesions which may include benign lesions [106, 108].

Two studies demonstrated the efficacy of the application of ICG intraoperatively for the identification of bile leakage following hepatic resection. The RCT by Kaibori et al. showed no post-operative bile leaks when evaluating with fluorescence while the standard leak test without fluorescence had 10% leak rate. Marino et al. in their case matched study found that ICG fluorescence was able to identify bile leaks in 12% of patients at the liver surface from resection; leaks were promptly sutured, and subsequently had no development of post-operative leaks [108, 114].

*Statements*

i. ICG fluorescence-guided liver surgery can be useful for identifying more small superficial liver tumours (within 10 mm from the liver surface) compared to conventional imaging (LoE: Moderate).
ii. ICG fluorescence-guided liver surgery can be useful to enhance the identification of deeper tumours during dissection (LoE: Low)
iii. ICG fluorescence-guided liver surgery for primary liver tumours may help to achieve a better resection margin in comparison to intraoperative Ultrasound (IOUS) (LoE: Low).
iv. ICG fluorescence-guided detection of liver lesions may result in a false positive rate of up to 25% (LoE: Moderate).
v. ICG fluorescence is useful for intraoperative detection and prevention of bile leaks from the cut liver surface when ICG is injected through the biliary tree (LoE: Strong).

*Recommendations*

We recommend the use of ICG fluorescence in liver surgery to aid identification of bile leaks after liver resection.

Grade of recommendation: Weak.

This recommendation received a 71% agreement on first round online survey.

We recommend the use of IOUS during liver surgery to complement the accuracy of ICG fluorescence newly detected lesions.

Grade of recommendation: Strong.

This recommendation received a 71% agreement on first round online survey.

The use of ICG fluorescence in liver surgery may improve detection of superficial liver tumours.

Grade of recommendation: Strong.

This recommendation received a 72% agreement on second-round online survey.

We recommend the use of ICG fluorescence in liver surgery may improve R0 resection rate for hepatic lesions.

Grade of recommendation: Weak.

This recommendation received a 62% agreement on second-round online survey.

### ICG Fluorescence for perfusion assessment in Upper GI surgery

As well as for colorectal surgery, ensuring good visceral perfusion is probably the most important controllable factor in preventing anastomotic leakage. ICG fluorescent angiography has also been investigated for esophagectomy and gastrectomy, especially for perfusion assessment of the gastric conduit during esophagectomy, as the perfusion of the tube, especially in the proximal part, is solely based on the right gastroepiploic artery. ICG fluorescence might guide surgeons in estimating the blood supply of the gastric segment and identifying the optimal anastomotic site [114].

The literature search identified 188 abstracts. After screening, 30 papers were assessed for eligibility, although many were excluded being case reports or pilot studies on few patients. 9 studies were included in the final data analysis [115–123]. No RCTs were found, and 5 prospective and 4 retrospective studies were analysed. In all papers, ICG fluorescent angiography was applied to assess gastric conduit perfusion in minimally invasive Ivor-Lewis esophagectomies. Anastomotic leak (AL) rate has been evaluated as the primary outcome by all authors: five studies were designed as propensity score case-match comparison with historical series of standard esophagectomies; in all cases, AL rate was decreased in the ICG group compared to standard light vision (in 3 studies with statistical significance). These data were cross-referenced and confirmed by a late 2019 meta-analysis on the topic, where six trials that compared ICG fluorescence perfusion assessment with standard technique cases showed an AL rate risk reduction of 69% [124]. Most studies reported a change of strategy on the planned anastomotic site, up to 25% of cases, when ICG fluorescence was considered unsatisfactory; as regards perfusion evaluation, most recent studies also reported, as secondary outcomes, data on quantitative assessment of perfusion (especially in terms of evaluation of fluorescence intensity or time until acceptable subjective fluorescence was documented on the conduit).

*Statement*

The use of ICG fluorescence to assess tissue perfusion may be effective in reducing the risk of a leak in esophago-gastric anastomosis (LoE: moderate/low).

*Recommendations*

The use of ICG fluorescence is recommended to assess tissue perfusion in order to reduce the risk of anastomotic leak in esophago-gastric anastomosis.

Grade of recommendation: Weak.

This recommendation received a 72% agreement in the first round of the online survey.

We recommend further research on the quantitative evaluation of ICG fluorescence in order to reduce subjective variability in perfusion assessment.

Grade of recommendation: Strong.

This recommendation received a 93% agreement in the first round of the online survey.

### ICG Fluorescence for lymphatic mapping in Upper GI surgery

As already mentioned in addressing the role of ICG fluorescent lymphography for colorectal cancer, the possibility of real-time navigation of lymph nodes and lymphatic routes appears to be of great interest even more in Upper GI malignancies and might have significant clinical consequences. Especially for gastric cancer, several studies are available demonstrating that ICG is superior to both radioactive tracers and other probes used to date, showing high sensitivity in identifying not a single sentinel node but a group of lymph nodes and lymphatic channels that represents the first drainage stations from the tumour, which has been referred as the lymphatic basin. Over the years, the lymphatic basin concept has been investigated, especially in relation to early gastric cancer, focussing on ICG fluorescence lymphatic mapping aiming to customise surgical lymphadenectomy according to tumour T stage, patient condition and risk profile [125].

A total of 553 records were identified. Following screening for eligibility and according to inclusion/exclusion criteria established, 7 articles were included in the final analysis: 1 RCT, 4 prospective and 2 retrospective studies [126–132]. All studies evaluated ICG fluorescence lymphatic mapping for gastric cancer: in four studies, including the RCT, lymphography was performed in laparoscopic gastrectomy (both distal and D2 total gastrectomies), while in the other three prospective studies, surgical procedures were robotics. ICG injection in the peritumoral area was performed endoscopically in the submucosal layer in all studies, either intraoperatively or the day before surgery. The main outcome analysed was the number of removed lymph nodes; 5 studies, including the RCT conducted on 260 patients, demonstrated that ICG lymphatic mapping could noticeably improve lymphadenectomy (higher number of lymph nodes retrieved compared to white light standard imaging technique). No significant differences in post-operative complications were reported between the two techniques. Peri-operative outcomes were also reported as secondary outcomes in all papers where 2 studies demonstrated that ICG lymphography was significantly effective in reducing operative time and intraoperative blood loss compared to a standard light.

*Statement*

During gastric cancer surgery, ICG fluorescent lymphatic mapping by endoscopic injection before surgery is safe and feasible and may lead to the identification and removal of a higher number of lymph nodes (LoE: moderate).

*Recommendation*

The use of ICG fluorescent lymphatic mapping during gastric cancer surgery may be recommended to improve lymphadenectomy.

Grade of recommendation: Strong.

This recommendation received an 84% agreement in the second-round of the online survey.

### Gynaecologic surgery

ICG fluorescence-guided imaging in gynaecologic surgery is used primarily for sentinel node dissection in endometrial and cervical cancer: indeed, accurate identification of sentinel lymph nodes in patients with cancer improves the detection of metastatic disease, and might decreases surgical morbidity. In this field, ICG lymphography has already proven to be a feasible, safe, time-efficient and reliable method for lymphatic mapping, with better bilateral detection rates; it would also avoid patients’ exposure to radioactive tracers, and for this reason, in some countries, ICG sentinel node mapping has already become the gold standard. Experience in vulvar cancer is more limited, with ICG used together with Tc-99 m as a dual tracer and alone in video endoscopic inguinal lymphadenectomy, while in early ovarian cancer, results are still preliminary but promising [133].

A total of 4260 records were identified. Following abstract screening for eligibility, 28 full texts were included for potential data extraction and assessment of the risk of bias. However, given the number and quality of studies found, 15 articles were finally included in the qualitative analysis: 2 RCTs and 13 prospective studies [134–148]. Both RCTs were comparing ICG versus methylene blue in sentinel nodes detection in cervical and uterine cancer; in particular, the FILM trial, published in the Lancet Oncology in 2018, was designed as a non-inferiority trial but ended up demonstrating that ICG mapping was superior to standard blue dye, being able to identify sentinel lymph nodes in a much larger proportion of patients, to detect at least one sentinel node and more effective in bilateral sentinel nodes identification.

It also has to be mentioned that in this setting the research and article screening was not conducted by a team of gynaecologists, however the analysis of the articles included and the creation of the statements was strongly based on a systematic review and consensus statement paper recently published on Annals of Surgical Oncology [133].

During the online survey, less than 60% of EAES surgeon members showed agreement on this topic, while almost 40% of them gave a “don’t know/no opinion” answer; for this reason, the expert panel decided not to run a second-round survey on this topic: since EAES members are mostly general/abdominal surgeons, we present hereby literature search results and expert’s discussion result, although no consensus was reached on Gynaecologic surgery setting.

*Statements*

i. In surgery for endometrial, cervical and vulvar cancer, ICG fluorescent lymphatic mapping for sentinel node dissection and lymph nodes detection is safe and feasible (LoE: strong).
ii. In surgery for endometrial, cervical and vulvar cancer, ICG fluorescent lymphatic mapping for sentinel node dissection and lymph nodes detection can be as effective as radioactive tracers and more effective than other dye tracers (LoE: strong)

*Recommendation*

We recommend the use of ICG fluorescence lymphatic mapping during surgery for endometrial and vulvar cancer.

Grade of recommendation: Strong.

This recommendation received a 50% agreement on the first round of the online survey. No second-round survey has been performed for the above-mentioned reasons.

### Urologic surgery

As regards urologic surgery, ICG fluorescence imaging has been largely explored since this technology became available in robotic systems, which are widely employed in this surgical speciality. ICG fluorescence has been found to be useful during robotic partial nephrectomy in guiding selective/super-selective clamping of arteries, while differential fluorescence intensity may play a role in discerning between pathological and normal renal tissue resulting in the minimal renal parenchymal loss (only feasibility and preliminary studies available on this latter application). ICG guidance during robotic radical prostatectomy and cystectomy has been found to better-assist surgeons in identifying lymphatic drainage both for sentinel lymph node biopsy and for extended lymph node dissection, where several studies have shown, as for gastrointestinal and gynaecological tumours, a higher number of lymph nodes removed compared to the standard white light imaging [149].

A total of 394 records were identified. Following abstract screening for eligibility, 27 full texts were included for potential data extraction and assessment of the risk of bias. However, given the number and quality of studies found, 19 articles were finally included in qualitative analysis: 1 RCT, 13 prospective series, mainly with historic case-match comparison, and 5 large retrospective studies [150–168]. In eight studies, including the RCT, the object was robotic radical prostatectomy demonstrating that the use of ICG fluorescence imaging during extended pelvic lymph node dissection improves the identification of lymphatic drainage and tissue, resulting in a higher yield of lymph nodes compared to standard vision.

The other eleven studies analysed the role of fluorescence imaging in robotic partial nephrectomy, where ICG has been used to clarify vascular anatomy to perform selective clamping of the tumour-feeding vascular branches aiming to reduce ischemic renal trauma and potentially improve kidney function preservation. All studies reported that this procedure is safe and feasible and potentially leads to short-term renal functional outcomes.

As happened for the “gynaecology setting”, during the online survey, less than 60% of EAES surgeon members showed agreement on this topic, while almost 40% of them gave a “don’t know/no opinion” answer; for this reason, the expert panel decided not to run a second-round survey on this topic: since EAES members are mostly general/abdominal surgeons, we present hereby literature search results and expert’s discussion result, although no consensus was reached on Urologic surgery.

Also, for this topic, it is worth reporting that there is a large number of studies regarding ICG fluorescence-guided surgery applied to multiple fields and different surgical procedures and that in April 2020, it was published in the World Journal of Urology, an extensive systematic literature review to provide evidence-based expert recommendations on best practices in this field, to which we referred in our analysis [149, 169].

*Statements*

i. ICG fluorescent lymphatic mapping for sentinel node dissection and lymph nodes detection during prostatectomy and cystectomy for cancer is safe and feasible (LoE: high).
ii. ICG fluorescence lymphatic mapping in radical prostatectomy may lead to the identification and removal of a higher number of lymph nodes (LoE: moderate).
iii. ICG fluorescence-guided robotic partial nephrectomy may offer better short-term renal functional outcomes by favouring selective clamping as compared to standard partial nephrectomy (LoE: low).
iv. There is insufficient evidence to support the application of ICG fluorescence during robotic partial nephrectomy to differentiate renal tumours from normal kidney parenchyma (LoE: low).

*Recommendations*

We recommend the use of ICG fluorescent lymphatic mapping during radical prostatectomy for the removal of a higher number of lymph nodes.

Grade of recommendation: Weak.

We recommend further research on the use of ICG fluorescence in urologic surgery to assess its potential clinical benefits.

Grade of recommendation: Strong.

This recommendation received a 50% agreement on the first round of the online survey. No second-round survey has been performed for the above-mentioned reasons.

### Ongoing trials

At the time of writing, searching registries of privately and publicly funded clinical studies for the terms “minimally invasive surgery”, “laparoscopy”, “robotic” and “fluorescence” we found 17 ongoing trial registered on ClinicalTrials.gov: 6 in Europe, 3 in the United States, 2 in Asia, 1 in Turkey, one in South America, two in North America. As regards study design, two monocentric randomized controlled trials (RCTs), two multicentric RCTs, 13 monocentric observational trials are registered.

Four of them haven’t started recruiting yet. The remaining 13 trial are still recruiting (estimated studies completion date 2022–2024).

13 studies concern laparoscopic surgery, 4 the robotics. The main focus is oncological surgery (Upper GI, colorectal, prostate, hepatobiliary and lung cancer, peritoneal carcinomatosis, liver resection). Two non-oncological studies are focussed on hepatobiliary surgery and one more on minimally invasive general surgery.

Primary outcomes of the studies are: feasibility of ICG fluorescence imaging in laparoscopic and robotic surgery, the usefulness of ICG to guide lymphadenectomy in oncological surgery, enhanced anatomical visualization, primary tumour detection, localization of occult lesions, anastomotic leak prevention. Common secondary outcomes are: impact of ICG on perioperative complications, side effects after indocyanine green injection, surgical time, conversion rate, surgeon confidence, hospital stay.

## Discussion

### What is new in this Consensus paper?

This is the first Consensus on ICG fluorescence-guided surgery edited by the EAES. It covered the application of this technology to several different districts of interest, including urology and gynaecology. Compared to other guidelines available in the literature, this represents the literature-based opinion of a large group of endoscopic surgeons since the systematic analysis of the literature by the experts panel was followed by a two-rounds online survey extended to all EAES members.

### Implementation

The Consensus believes that it is feasible to successfully implement these recommendations into local practice and that the recommendations will be accepted by stakeholders. The main considerations regarding the implementation of this Consensus include costs and availability of the technology. In addition, some of the recommended techniques require specialized knowledge and skills. Finally, in order to achieve the full benefit of these recommendations, it is advised to standardize the techniques, for what it entails dose, concentration and route and timing of administrations of ICG depending on the different applications. The panel plans to survey physicians in the future in order to monitor and audit compliance with the recommendations put forth in this Consensus.

### Updating this Consensus

The EAES plans to repeat a comprehensive literature review in three years to reevaluate and identify new evidences. Particular attention will be paid to any future studies that specifically address the research recommendations proposed in this Consensus. A formal update will be generated when substantial literature is detected. When sufficient literature is available, the EAES will project to produce a to produce a structured guideline with summary evidence appraisal and a formal evidence-to-decision framework.

### Limitations of this Consensus

The main limitation of this Consensus is the low certainty of evidence for some of the key questions. In addition, being a Consensus, patients’ values were not actually obtained. On the contrary, the panel’s impression of their beliefs was used, based on experiences with patients. While the recommendations in this Consensus are based on the highest-level evidence meeting inclusion criteria, cost-effectiveness was not specifically addressed. Moreover, we were not able to take into account certain aspects of diversity, equity, and inclusion due to unavailability in the literature that was reviewed.

## Conclusions

The consensus conference proposed a wide number of recommended applications of ICG fluorescence-guided surgery aiming to patients’ benefit in different surgical specialties. These evidence-based recommendations are aimed to support safe diffusion of the technology. Whilst there are clear and strong evidence in certain areas to support its safety and effectiveness in improving clinical outcomes, further robust studies are required to improve the standardization of the techniques and to explore different possible applications.

## Supplementary Information

Below is the link to the electronic supplementary material.

Supplementary file1 (PDF 74 KB)

Supplementary file2 (PDF 113 KB)

Supplementary file3 (PDF 85 KB)

Supplementary file4 (PDF 110 KB)

Supplementary file5 (PDF 97 KB)

Supplementary file6 (PDF 113 KB)

Supplementary file7 (PDF 114 KB)

Supplementary file8 (PDF 114 KB)

Supplementary file9 (PDF 109 KB)

Supplementary file10 (PDF 85 KB)

Supplementary file11 (PDF 110 KB)

Supplementary file12 (PDF 81 KB)

Supplementary file13 (PDF 81 KB)

Supplementary file14 (PDF 109 KB)

Supplementary file15 (PDF 80 KB)

Supplementary file16 (PDF 124 KB)

Supplementary file17 (PDF 122 KB)

Supplementary file18 (PDF 108 KB)

Supplementary file19 (PDF 116 KB)

Supplementary file20 (PDF 130 KB)

Supplementary file21 (PDF 99 KB)

Supplementary file22 (PDF 112 KB)

Supplementary file23 (PDF 84 KB)

Supplementary file24 (PDF 147 KB)
