# Supplementary material for: European Association for Endoscopic Surgery (EAES) consensus on Indocyanine Green (ICG) fluorescence-guided surgery
Source: Surg Endosc. 2023 Feb 13;37(3):1629–48. doi: 10.1007/s00464-023-09928-5 (PMC10017637; doi:10.1007/s00464-023-09928-5)
Supplement: Supplementary file 1 — Supplementary file1 (PDF 74 KB) [file 464_2023_9928_MOESM1_ESM.pdf]

Surgery guided by ICG (Indocyanine green) enhanced fluorescence

Clinical question, PICOS and Search Strategy

Setting: BARIATRIC SURGERY

Clinical question: **Would indocyanine green - enhanced fluorescence surgery, rather than surgery without fluorescence - improve the outcome of patients after BARIATRIC SURGERY?**

P = Population or Patient group: patients who underwent standard, laparoscopic or robotic **BARIATRIC SURGERY**
I= Intervention: surgical procedure (standard, laparoscopic, robotic) with fluorescent properties of indocyanine green (ICG)
C= Comparator: surgical procedure (standard, laparoscopic, robotic) without fluorescent properties of indocyanine green (ICG)
O = Outcomes: mortality, morbidity**,** operating time, re-operation, re-admission
S = Study design

- Primary research: randomised controlled trials (RCTs), controlled cohort studies, case control studies
- Secondary research: systematic reviews and meta analysis

**Keyword A**	Bariatric surgery				
**Keyword B**	Fluorescence				
**Keyword C**	Green Indocyanine				
**Keyword C**	Outcome				
**Search strategy**	Bariatric surgery	**AND**	Fluorescence	**AND**	Green Indocyanine
AND	Outcome	**AND**	Morbidity		Mortality

Search methods for identification of studies: all sources searched, including: databases, trials registers, websites and grey literature; all types of studies included: case series, clinical trials, review and meta-analysys

Search strategy Pubmed

((("bariatric surgery"[MeSH Terms] OR ("bariatric"[All Fields] AND "surgery"[All Fields]) OR "bariatric surgery"[All Fields]))) AND (((((("Indocyanine Green"[Mesh] OR "Fluorescent Dyes"[Mesh] OR "indocyanine green" OR wofaverdin OR vophaverdin OR fluorescen* OR cw800*)) OR ("near infrared fluorescence" OR "near infrared fluoresce imaging"))))))))))

Search strategy Embase

((bariatric surgery/ or bariatric surgery.af.) or (bariatric.af. and surgery.af.)) and (indocyanine green/ or fluorescent dye/ or indocyanine green.af. or wofaverdin.af. or "fluorescen*".af. or "CW800*".af. or near infrared fluorescence.af.)
