# Supplementary material for: European Association for Endoscopic Surgery (EAES) consensus on Indocyanine Green (ICG) fluorescence-guided surgery
Source: Surg Endosc. 2023 Feb 13;37(3):1629–48. doi: 10.1007/s00464-023-09928-5 (PMC10017637; doi:10.1007/s00464-023-09928-5)
Supplement: Supplementary file 3 — Supplementary file3 (PDF 85 KB) [file 464_2023_9928_MOESM3_ESM.pdf]

Surgery guided by ICG (Indocyanine green) enhanced fluorescence

Clinical question, PICOS and Search Strategy

Setting: Cholecystectomy

Clinical question: **Would indocyanine green - enhanced fluorescence surgery, rather than surgery without fluorescence - improve the outcome of patients after cholecystectomy?**

P = Population or Patient group: patients who underwent standard, laparoscopic or robotic surgery **(cholecystectomy for symptomatic lithiasis or/and cholecystitis)**
I= Intervention: surgical procedure (standard, laparoscopic, robotic) with fluorescent properties of indocyanine green (ICG)
C= Comparator: surgical procedure (standard, laparoscopic, robotic) without fluorescent properties of indocyanine green (ICG)
O = Outcomes: mortality, morbidity**,** operating time, re-operation, re-admission
S = Study design

- Primary research: randomised controlled trials (RCTs), controlled cohort studies, case control studies
- Secondary research: systematic reviews and meta analysis

**Keyword A**	Indocyanine Green				
**Keyword B**	ICG				
**Keyword C**	Fluorescence Cholangiography				
**Keyword C**	Image-guided surgery		Cholecystectomy		
**Search strategy**	Indocyanine Green	**OR**	ICG	**OR**	Fluorescence Cholangiography
**OR**	Image-guided surgery	**AND**	Cholecystectomy		

Search methods for identification of studies: all sources searched, including: databases, trials registers, websites and grey literature; all types of studies included: case series, clinical trials, review and meta-analysys – English language only

Search Strategy Pubmed

((((cholecystectomy OR cholecystectomy[MeSH Major Topic])) OR (("Bile Ducts/surgery"[Mesh] OR "bile duct injury " OR "bile duct injuries")))) AND ((((((("Indocyanine Green"[Mesh] OR "Fluorescent Dyes"[Mesh] OR "indocyanine green" OR wofaverdin OR vophaverdin OR fluorescen* OR cw800*)) OR ("near infrared fluorescence" OR "near infrared fluoresce imaging"))))

Search Strategy Embase

('cholecystectomy'/exp OR 'bile duct surgery'/exp OR 'gallbladder surgery'/exp OR 'ileostomy'/exp OR 'bile duct injury'/exp OR chiìolecystect*) AND ('laparoscopy'/exp OR laparosc* OR laparoendosc* OR celioscop* OR 'minimally invasive surgery'/exp OR 'robotic surgical procedure'/exp))) AND ('indocyanine green'/exp OR 'fluorescence'/exp OR indocyan* OR wofaverdin*' OR fluorescen* OR fluorescein* OR icg OR icgfa OR 'fluorescent dye'/exp)
