# Supplementary material for: European Association for Endoscopic Surgery (EAES) consensus on Indocyanine Green (ICG) fluorescence-guided surgery
Source: Surg Endosc. 2023 Feb 13;37(3):1629–48. doi: 10.1007/s00464-023-09928-5 (PMC10017637; doi:10.1007/s00464-023-09928-5)
Supplement: Supplementary file 5 — Supplementary file5 (PDF 97 KB) [file 464_2023_9928_MOESM5_ESM.pdf]

Surgery guided by indocyanine green enhanced fluorescence

Clinical question, PICOS and Search Strategy

GYNECOLOGIC SURGERY

Clinical question: **Would indocyanine green - enhanced fluorescence surgery, rather than surgery without fluorescence - improve the outcome of patients after GYNECOLOGIC surgery?**

P = Population or Patient group: patients who underwent standard, laparoscopic or robotic **GYNECOLOGIC** surgery
I= Intervention: surgical procedure (standard, laparoscopic, robotic) with fluorescent properties of indocyanine green (ICG)
C= Comparator: surgical procedure (standard, laparoscopic, robotic) without fluorescent properties of indocyanine green (ICG)
O = Outcomes: mortality, morbidity**,** operating time, re-operation, re-admission
S = Study design

- Primary research: randomised controlled trials (RCTs), controlled cohort studies, case control studies
- Secondary research: systematic reviews and meta analysis

**Keyword A**	Indocyanine green				
**Keyword B**	fluorescent dyes				
**Keyword C**	near infreared fluorescence				
**Keyword C**	gynecologic surgical procedures				
**Search strategy**	Indocyanine green	**OR**	near infreared fluorescence	**OR**	fluorescent dyes
**AND**	gynecologic surgical procedures	**OR**	Endometrial cancer, uterine cancer, ovarian cancer		

Search methods for identification of studies: all sources searched, including: databases, trials registers, websites and grey literature; all types of studies included: case series, clinical trials, review and meta-analysys
English language only

Pubmed
((((("Indocyanine Green"[Mesh] OR "Fluorescent Dyes"[Mesh] OR “indocyanine green” OR wofaverdin OR vophaverdin OR fluorescen* OR cw800*)) OR ("near infrared fluorescence" OR "near infrared fluoresce imaging"))))) AND (("Gynecologic Surgical Procedures"[Mesh]) OR ("endometrial cancer" or "uterine cancer" or "endometriosis" or "ovariectomy" or " hysterectomy" or "sentinel lymph node"))

Embase
('indocyanine green'/exp OR 'fluorescent dyes'/exp OR 'indocyanine green' OR wofaverdin OR vophaverdin OR fluorescein* OR cw800* OR 'near infrared fluorescence' OR 'near infrared fluorescence imaging') AND ('gynecologic surgical procedures'/exp OR 'endometrial cancer' OR 'uterine cancer' OR 'endometriosis' OR 'ovariectomy' OR 'hysterectomy' OR 'sentinel lymph node')
