# Supplementary material for: European Association for Endoscopic Surgery (EAES) consensus on Indocyanine Green (ICG) fluorescence-guided surgery
Source: Surg Endosc. 2023 Feb 13;37(3):1629–48. doi: 10.1007/s00464-023-09928-5 (PMC10017637; doi:10.1007/s00464-023-09928-5)
Supplement: Supplementary file 8 — Supplementary file8 (PDF 114 KB) [file 464_2023_9928_MOESM8_ESM.pdf]

Surgery guided by indocyanine green enhanced fluorescence

Clinical question, PICOS and Search Strategy
Setting: LIVER SURGERY

Clinical question: **Would indocyanine green - enhanced fluorescence surgery, rather than surgery without fluorescence - improve the outcome of patients after liver resection?**

P = Population or Patient group: patients who underwent standard, laparoscopic or robotic surgery **(liver resection for hepatic lesions – primary or metastatic tumor)**
I= Intervention: surgical procedure (standard, laparoscopic, robotic) with fluorescent properties of indocyanine green (ICG)
C= Comparator: surgical procedure (standard, laparoscopic, robotic) without fluorescent properties of indocyanine green (ICG)
O = Outcomes: mortality, morbidity**,** operating time, re-operation, re-admission, identification of lesion, improving surgical procedures in term of radicality of intervention and reducing bile leakage
S = Study design

- Primary research: randomised controlled trials (RCTs), controlled cohort studies, case control studies
- Secondary research: systematic reviews and meta analysis

**Keyword A**	Indocyanine green			
**Keyword B**	Hepatic surgery			
**Keyword C**	Liver resection			
**Keyword C**	Fluorescence guided surgery			
**Search strategy**	Indocyanine green	**OR**	Fluorescence guided surgery	
**AND**	Hepatic surgery	**OR**	Liver resection	

Search methods for identification of studies: all sources searched, including: databases, trials registers, websites and grey literature; all types of studies included: case series, clinical trials, review and meta-analysys
English language only

Search strategy

Pubmed

((("Liver"[Mesh] OR hepatic*) AND (liver-metastases* OR "hepatic lesion" OR "hepatic resection" OR "hepatic neoplasm" OR "hepatic margin") AND ("surgery"[Subheading] OR surger* OR surgeo* OR surgi* OR resect* OR "Laparoscopy"[Mesh] OR laparosc* OR laparoendosc* OR celioscop* OR "Minimally Invasive Surgical Procedures"[Mesh] OR "Robotic Surgical Procedures"[Mesh]) AND ("Indocyanine Green"[Mesh] OR "Fluorescent Dyes"[Mesh] OR "indocyanine green" OR wofaverdin OR vophaverdin OR cw800*) OR ("near infrared fluorescence" OR "near infrared fluorescence imaging")))

Embase

(('Indocyanine Green' OR 'Fluorescent Dyes'/exp OR 'indocyanine green'/exp OR wofaverdin OR vophaverdin OR cw800*) OR ('near infrared fluorescence' OR 'near infrared fluorescence imaging')
AND (('liver'/exp OR 'hepatic'/exp) AND ('liver metastases' OR 'hepatic lesion' OR 'hepatic resection' OR 'hepatic neoplasm' OR 'hepatic margin')))
