# Supplementary material for: European Association for Endoscopic Surgery (EAES) consensus on Indocyanine Green (ICG) fluorescence-guided surgery
Source: Surg Endosc. 2023 Feb 13;37(3):1629–48. doi: 10.1007/s00464-023-09928-5 (PMC10017637; doi:10.1007/s00464-023-09928-5)
Supplement: Supplementary file 10 — Supplementary file10 (PDF 85 KB) [file 464_2023_9928_MOESM10_ESM.pdf]

Surgery guided by ICG (Indocyanine green) enhanced fluorescence

Clinical question, PICOS and Search Strategy
Setting: Lymphatic Mapping in Colorectal Surgery

Clinical question: **Would indocyanine green - enhanced fluorescence surgery, rather than surgery without fluorescence - improve the outcome of patients undergoing colorectal resection with lymphnodes removal?**

P = Population or Patient group: patients who underwent standard, laparoscopic or robotic surgery **colorectal surgery**
I= Intervention: surgical procedure (standard, laparoscopic, robotic) with fluorescent properties of indocyanine green (ICG)
C= Comparator: surgical procedure (standard, laparoscopic, robotic) without fluorescent properties of indocyanine green (ICG)
O = Outcomes: mortality, morbidity**,** operating time, re-operation, re-admission
S = Study design

- Primary research: <u>randomised controlled trials (RCTs), controlled cohort studies, case control studies</u>
- Secondary research: systematic reviews and meta analysis

**Keyword A**	Indocyanine Green				
**Keyword B**	ICG				
**Keyword C**	Fluorescence Lymphangiography/ Lymphography				
**Keyword C**	Image-guided surgery		Colorectal		
**Search strategy**	Indocyanine Green	**OR**	ICG	**OR**	Fluorescence Lymphangiograp hy/Lymphograph
**OR**	Image-guided surgery	**AND**	Colorectal surgery		

<u>Search methods for identification of studies:</u> all sources searched, including: databases, trials registers, websites and grey literature; all types of studies included: case series, clinical trials, review and meta-analysys – <u>English language only</u>

Search Strategy Pubmed

(("Colorectal Surgery"[Mesh] OR “Colectomy”[Mesh] OR colectom* OR polypect* OR bowel-resect* OR ((“Colon”[Mesh] OR “Colon Cancer”[Mesh] OR “Rectum”[Mesh] OR “Rectal Cancer”[Mesh] OR colon* OR colonic* OR rectum* OR rectal* OR colorect* OR colo-rect* OR polyposis-coli) AND ("surgery"[Subheading] OR surger* OR surgeo* OR surgi* OR resect* OR "Laparoscopy"[Mesh] OR laparosc* OR laparoendosc* OR celioscop* OR "Minimally Invasive Surgical Procedures"[Mesh] OR "Robotic Surgical Procedures"[Mesh]))) AND ("Indocyanine Green"[Mesh] OR "Fluorescence"[Mesh] OR indocyan* OR indo-cyan* OR fluorescen* OR fluorescein* OR ICG OR dye* OR “Fluorescent Dyes”[Pharmacological Action] OR "Fluorescein Angiography"[Mesh]) AND ("Lymphadenectomy"[Mesh] OR "Lymphatic Mapping"[Mesh] OR “Sentinel Lymphnode”[Mesh] OR lymphnod* OR lymph-nod* OR lymphectomy* OR "Lymphography"[Mesh] OR lymphogr*))

Search Strategy Embase

('colorectal surgery'/exp OR 'colon surgery'/exp OR 'colectomy'/exp OR 'polypectomy'/exp OR colectom* OR 'bowel resect*' OR (('colon'/exp OR 'colon disease'/exp OR 'rectum'/exp OR 'rectum disease'/exp OR colon OR colonic* OR rectum* OR rectal* OR colorect* OR 'colo rect*' OR 'polyposis coli' OR sigmoid*) AND ('surgery':lnk OR surger* OR surgeo* OR surgi* OR resect* OR 'laparoscopy'/exp OR laparosc* OR laparoendosc* OR celioscop* OR 'minimally invasive surgery'/exp OR 'robotic surgical procedure'/exp))) AND ('indocyanine green'/exp OR 'fluorescence'/exp OR indocyan* OR 'indo cyan*' OR fluorescen* OR fluorescein* OR icg OR icgfa OR 'coloring agent'/exp OR colouring OR coloring OR dye* OR 'fluorescent dye'/exp OR 'fluorescence angiography'/exp) AND ('lymphadenectomy'/exp OR 'lymphatic mapping'/exp OR lymphectomy* OR lymphography* OR 'sentinel lymphnode'/exp OR lymphnod* OR ‘lymphography’/exp OR lymphogr*)
