# Supplementary material for: European Association for Endoscopic Surgery (EAES) consensus on Indocyanine Green (ICG) fluorescence-guided surgery
Source: Surg Endosc. 2023 Feb 13;37(3):1629–48. doi: 10.1007/s00464-023-09928-5 (PMC10017637; doi:10.1007/s00464-023-09928-5)
Supplement: Supplementary file 12 — Supplementary file12 (PDF 81 KB) [file 464_2023_9928_MOESM12_ESM.pdf]

Surgery guided by indocyanine green enhanced fluorescence
Clinical question, PICOS and Search Strategy
Setting: Lymphatic Mapping in UpperGI Surgery

Clinical question: **Would indocyanine green - enhanced fluorescence surgery, rather than surgery without fluorescence - improve lymphadenectomy/lymphnode mapping and retrieval in patients undergoing esophagogastric surgery:**

- Minimally Invasive esophagectomy with esophagogastric anastomosis
- Minimally Invasive total or partial gastrectomy

P = Population or Patient group: patients who underwent laparoscopic or robotic esophagogastric surgery for esophageal, esophagogastric junction or gastric cancer

I= Intervention: esophagectomy with esophagogastric anastomosis and gastrectomy (laparoscopic, robotic) with fluorescent properties of indocyanine green (ICG)

C= Comparator: open esophagectomy with esophagogastric anastomosis and open gastrectomy without fluorescent properties of indocyanine green (ICG)

O = Outcomes:

Primary outcomes of interest were identification rate, sensitivity, specificity, and the presence of lymphnode of patients with esophageal/gastric cancer. Secondary outcomes: mortality, morbidity, long term outcomes

S = Study design

- o Primary research: randomised controlled trials (RCTs), controlled cohort studies, case control studies
- o Secondary research: systematic reviews and meta analysis

**Keyword A**	esophageal cancer or esophagogastric junction cancer - gastric/stomach				
**Keyword B**	indocyanine green (ICG) fluorescence lymphography/lymphatic mapping				
**Keyword C**	minimally invasive surgery - Ivor Lewis esophagectomy – laparoscopic gastrectomy				
**Search strategy**	indocyanine green (ICG)	**OR**	Near-infrared fluorescence/ sentinel lymph node detection/ gastric cancer	OR	Fluorescence Lymphangiography/Lymphatic mapping
**AND**	esophageal cancer or esophagogastric junction cancer - gastric/stomach	**OR**	minimally invasive surgery - Ivor Lewis esophagectomy – laparoscopic gastrectomy		

Search methods for identification of studies: all sources searched, including: databases, trials registers, websites and grey literature; all types of studies included: case series, clinical trials, review and meta-analysys - **English language only**

Search Strategy

Pubmed

(("Gastric Surgery"[Mesh] OR "Gastrectomy"[Mesh] OR gastric-surgery* OR *gastric-resect* OR "Esophageal Surgery"[Mesh] OR esophagect* OR esophagogastrectomy* OR (("Stomach"[Mesh] OR

“Gastric Cancer”[Mesh] OR “Esophagus”[Mesh] OR “Esophageal Cancer”[Mesh] OR esophago-gastric* OR “Early gastric cancer” OR EGC*) AND ("surgery"[Subheading] OR surger* OR surgeo* OR surgi* OR resect* OR "Laparoscopy"[Mesh] OR laparosc* OR laparoendosc* OR celioscop* OR "Minimally Invasive Surgical Procedures"[Mesh] OR "Robotic Surgical Procedures"[Mesh]))) AND ("Indocyanine Green"[Mesh] OR "Fluorescence"[Mesh] OR indocyan* OR indo-cyan* OR fluorescen* OR fluorescein* OR ICG OR “Coloring Agents”[Mesh] OR “Coloring Agents”[Pharmacological Action] OR colouring OR coloring OR dye* OR “Fluorescent Dyes”[Pharmacological Action] OR "Fluorescein Angiography"[Mesh]) AND ("Lymphadenectomy"[Mesh] OR "Lymphatic Mapping"[Mesh] OR “Sentinel Lymphnode”[Mesh] OR “Lymphatic Basin” OR sentinel-lymph* OR lymphnod* OR lymph-nod* OR lymphectomy* OR "Lymphography"[Mesh] OR lymphogr*))

Embase

('gastric surgery'/exp OR 'gastrectomy'/exp OR 'esophageal surgery'/exp OR 'esophagogastrectomy'/exp OR gastric resect* OR 'esophagect*' OR (('stomach'/exp OR 'gastric cancer'/exp OR 'esophagus'/exp OR 'esophageal cancer'/exp OR esophago-gastric OR early gastric cancer*) AND ('surgery':lnk OR surger* OR surgeo* OR surgi* OR resect* OR 'laparoscopy'/exp OR laparosc* OR laparoendosc* OR celioscop* OR 'minimally invasive surgery'/exp OR 'robotic surgical procedure'/exp))) AND ('indocyanine green'/exp OR 'fluorescence'/exp OR indocyan* OR 'indo cyan*' OR fluorescen* OR fluorescein* OR icg OR icgfa OR 'coloring agent'/exp OR colouring OR coloring OR dye* OR 'fluorescent dye'/exp OR 'fluorescence angiography'/exp) AND ('lymphadenectomy'/exp OR 'lymphatic mapping'/exp OR ‘sentinel lymphnode’/exp OR ‘lymphatic basin’/exp OR sentinel-lymph* OR lymphectomy* OR lymphography* OR 'sentinel lymphnode'/exp OR lymphnod* OR ‘lymphography’/exp OR lymphogr*)
