# Supplementary material for: European Association for Endoscopic Surgery (EAES) consensus on Indocyanine Green (ICG) fluorescence-guided surgery
Source: Surg Endosc. 2023 Feb 13;37(3):1629–48. doi: 10.1007/s00464-023-09928-5 (PMC10017637; doi:10.1007/s00464-023-09928-5)
Supplement: Supplementary file 13 — Supplementary file13 (PDF 81 KB) [file 464_2023_9928_MOESM13_ESM.pdf]

Surgery guided by indocyanine green enhanced fluorescence

Clinical question, PICOS and Search Strategy

Setting: Pancreatic surgery

Clinical question: **Would indocyanine green - enhanced fluorescence surgery, rather than surgery without fluorescence - improve the outcome of patients after pancreatic surgery?**

P = Population or Patient group: patients who underwent standard, laparoscopic or robotic surgery **(Pancreatic surgery for adenocarcinoma and neuroendocrine tumor - NET)**
I= Intervention: surgical procedure (standard, laparoscopic, robotic) with fluorescent properties of indocyanine green (ICG)
C= Comparator: surgical procedure (standard, laparoscopic, robotic) without fluorescent properties of indocyanine green (ICG)
O = Outcomes: mortality, morbidity**,** operating time, re-operation, re-admission
S = Study design

- Primary research: randomised controlled trials (RCTs), controlled cohort studies, case control studies
- Secondary research: systematic reviews and meta analysis

**Keyword A**	Indocyanine green				
**Keyword B**	ICG				
**Keyword C**	Near-infrared fluorescence				
**Keyword C**	Pancreas surgery	Pancreas resection	Pancreas Tumor		Pancreas neuroendocrine tumor
**Search strategy**	Indocyanine green	**OR**	ICG	**OR**	Near-infrared fluorescence
AND	Pancreas surgery	**OR**	Pancreas resection	**OR**	Pancreas tumor
	Pancreas neuroendocrine tumor				

Search methods for identification of studies: all sources searched, including: databases, trials registers, websites and grey literature; all types of studies included: case series, clinical trials, review and meta-analysys
English language only

Search strategy

Pubmed:

(((("Pancreatic Diseases/surgery"[Mesh]) OR pancrea*) OR pancreas)) AND ((((("Indocyanine Green"[Mesh] OR "Fluorescent Dyes"[Mesh] OR “indocyanine green” OR wofaverdin OR vophaverdin OR cw800*)) OR ("near infrared fluorescence" OR "near infrared fluorescence imaging"))))))

Cochrane library

("Pancreatic Diseases/surgery" OR pancrea* OR pancreas) AND ("Indocyanine Green" OR "Fluorescent Dyes" OR "indocyanine green" OR wofaverdin OR vophaverdin OR cw800* OR "near infrared fluorescence" OR "near infrared fluorescence imaging")

Embase

('fluorescent dyes' OR 'indocyanine green'/exp OR 'indocyanine green' OR 'near infrared fluorescence'/exp OR 'near infrared fluorescence' OR 'near infrared fluorescence imaging'/exp) AND ('pancreas'/exp OR 'pancreas' OR 'pancreatic surgery' OR 'pancrea')
