# Supplementary material for: European Association for Endoscopic Surgery (EAES) consensus on Indocyanine Green (ICG) fluorescence-guided surgery
Source: Surg Endosc. 2023 Feb 13;37(3):1629–48. doi: 10.1007/s00464-023-09928-5 (PMC10017637; doi:10.1007/s00464-023-09928-5)
Supplement: Supplementary file 15 — Supplementary file15 (PDF 80 KB) [file 464_2023_9928_MOESM15_ESM.pdf]

Surgery guided by indocyanine green enhanced fluorescence
Clinical question, PICOS and Search Strategy
Setting: Perfusion Assessment in Colorectal Surgery

Clinical question: **Would indocyanine green - enhanced fluorescence surgery, rather than surgery without fluorescence - improve anastomotic leak rate through real time angiography and visceral perfusion assessment in patients undergoing colorectal surgery:**

P = Population or Patient group: patients who underwent standard, laparoscopic or robotic surgery **colorectal surgery**
I= Intervention: surgical colorectal procedure (standard, laparoscopic, robotic) with fluorescent properties of indocyanine green (ICG)
C= Comparator: open esophagectomy with esophagogastric anastomosis and open gastrectomy without fluorescent properties of indocyanine green (ICG)
O = Outcomes:
Primary outcome: anastomotic leak rate. Secondary outcomes: mortality, morbidity, length of hospital stay, long term outcomes
S = Study design
- o Primary research: randomised controlled trials (RCTs), controlled cohort studies, case control studies
- o Secondary research: systematic reviews and meta analysis

**Keyword A**	colorectal cancer or colorectal surgery				
**Keyword B**	indocyanine green (ICG) fluorescence angiography/perfusion assessment/vascularization assessment				
**Keyword C**	minimally invasive surgery - laparoscopic colorectal surgery				
**Search strategy**	indocyanine green (ICG)	**OR**	fluorescence angiography/perfusion assessment/vascularization assessment	**OR**	Anastomotic leak – anastomotic leakage
**AND**	colorectal cancer or colorectal surgery	**OR**	minimally invasive surgery - laparoscopic colorectal surgery		

Search methods for identification of studies: all sources searched, including: databases, trials registers, websites and grey literature; all types of studies included: case series, clinical trials, review and meta-analysys - **English language only**

Search Strategy

Pubmed
(("Colorectal Surgery"[Mesh] OR “Colectomy”[Mesh] OR “Ileostomy”[Mesh] OR “Colostomy”[Mesh] OR colectom* OR ileostom* OR colostom* OR polypect* OR bowel-resect* OR ((“Colon”[Mesh] OR “Colonic Diseases”[Mesh] OR “Rectum”[Mesh] OR “Rectal Diseases”[Mesh] OR colon OR colonic* OR rectum* OR rectal* OR colorect* OR colo-rect* OR polyposis-coli OR sigmoid*) AND ("surgery"[Subheading] OR surger* OR surgeo* OR surgi* OR resect* OR "Laparoscopy"[Mesh] OR laparosc* OR laparoendosc* OR celioscop* OR "Minimally Invasive Surgical Procedures"[Mesh] OR "Robotic Surgical Procedures"[Mesh]))) AND

("Indocyanine Green"[Mesh] OR "indocyanine green-sulfo-OSu"[Supplementary Concept] OR "Fluorescence"[Mesh] OR indocyan* OR indo-cyan* OR fluorescen* OR fluorescein* OR ICG OR ICGFA OR “Coloring Agents”[Mesh] OR “Coloring Agents”[Pharmacological Action] OR colouring OR coloring OR dye* OR “Fluorescent Dyes”[Pharmacological Action] OR "Fluorescein Angiography"[Mesh]) AND ("Anastomosis, Surgical"[Mesh] OR "Anastomotic Leak"[Mesh] OR anastomo* OR leak* OR "Perfusion"[Mesh] OR perfus* OR “blood supply”[Subheading] OR blood-suppl*))

Embase

('colorectal surgery'/exp OR 'colon surgery'/exp OR 'rectum surgery'/exp OR 'ileostomy'/exp OR 'polypectomy'/exp OR colectom* OR ileostom* OR colostom* OR polypect* OR 'bowel resect*' OR (('colon'/exp OR 'colon disease'/exp OR 'rectum'/exp OR 'rectum disease'/exp OR colon OR colonic* OR rectum* OR rectal* OR colorect* OR 'colo rect*' OR 'polyposis coli' OR sigmoid*) AND ('surgery':lnk OR surger* OR surgeo* OR surgi* OR resect* OR 'laparoscopy'/exp OR laparosc* OR laparoendosc* OR celioscop* OR 'minimally invasive surgery'/exp OR 'robotic surgical procedure'/exp))) AND ('indocyanine green'/exp OR 'fluorescence'/exp OR indocyan* OR 'indo cyan*' OR fluorescen* OR fluorescein* OR icg OR icgfa OR 'coloring agent'/exp OR colouring OR coloring OR dye* OR 'fluorescent dye'/exp OR 'fluorescence angiography'/exp) AND ('anastomosis'/exp OR 'anastomosis leakage'/exp OR anastomo* OR leak* OR 'perfusion'/exp OR perfus* OR 'blood suppl*')
