# Supplementary material for: European Association for Endoscopic Surgery (EAES) consensus on Indocyanine Green (ICG) fluorescence-guided surgery
Source: Surg Endosc. 2023 Feb 13;37(3):1629–48. doi: 10.1007/s00464-023-09928-5 (PMC10017637; doi:10.1007/s00464-023-09928-5)
Supplement: Supplementary file 16 — Supplementary file16 (PDF 124 KB) [file 464_2023_9928_MOESM16_ESM.pdf]

Surgery guided by indocyanine green enhanced fluorescence
Clinical question, PICOS and Search Strategy
Setting: Perfusion Assessment in UpperGI Surgery

Clinical question: **Would indocyanine green - enhanced fluorescence surgery, rather than surgery without fluorescence - improve anastomotic leak rate through real time angiography and visceral perfusion assessment in patients undergoing esophagogastric surgery:**

- Minimally Invasive esophagectomy with esophagogastric anastomosis
- Minimally Invasive total or partial gastrectomy

P = Population or Patient group: patients who underwent laparoscopic or robotic esophagogastric surgery for esophageal, esophagogastric junction or gastric cancer

I= Intervention: esophagectomy with esophagogastric anastomosis and gastrectomy (laparoscopic, robotic) with fluorescent properties of indocyanine green (ICG)

C= Comparator: open esophagectomy with esophagogastric anastomosis and open gastrectomy without fluorescent properties of indocyanine green (ICG)

O = Outcomes:

Primary outcome: anastomotic leak rate. Secondary outcomes: mortality, morbidity, length of hospital stay, long term outcomes

S = Study design

- o Primary research: randomised controlled trials (RCTs), controlled cohort studies, case control studies
- o Secondary research: systematic reviews and meta analysis

**Keyword A**	esophageal cancer or esophagogastric junction cancer - gastric/stomach				
**Keyword B**	indocyanine green (ICG) fluorescence angiography/perfusion assessment/vascularization assessment				
**Keyword C**	minimally invasive surgery - Ivor Lewis esophagectomy – laparoscopic gastrectomy				
**Search strategy**	indocyanine green (ICG)	**OR**	fluorescence angiography/perfusion assessment/vascularization assessment	**OR**	Anastomotic leak – anastomotic leakage
**AND**	esophageal cancer or esophagogastric junction cancer - gastric/stomach	**OR**	minimally invasive surgery - Ivor Lewis esophagectomy – laparoscopic gastrectomy		

Search methods for identification of studies: all sources searched, including: databases, trials registers, websites and grey literature; all types of studies included: case series, clinical trials, review and meta-analaysys - **English language only**

Search Strategy

Pubmed

(("Gastric Surgery"[Mesh] OR “Gastrectomy”[Mesh] ORgastric-surgery* OR gastric-resect* OR “Esophageal Surgery”[Mesh] OR esophagect* OR esophagogastrectomy* OR ((“Stomach”[Mesh] OR “Gastric Cancer”[Mesh] OR “Esophagus”[Mesh] OR “Esophageal Cancer”[Mesh] OR esophago-gastric* OR “Early gastric cancer” OR EGC*) AND ("surgery"[Subheading] OR surger* OR surgeo* OR surgi* OR resect* OR "Laparoscopy"[Mesh] OR laparosc* OR laparoendosc* OR celioscop* OR "Minimally Invasive Surgical Procedures"[Mesh] OR "Robotic Surgical Procedures"[Mesh]))) AND ("Indocyanine Green"[Mesh] OR "indocyanine green-sulfo-OSu"[Supplementary Concept] OR "Fluorescence"[Mesh] OR indocyan* OR indo-cyan* OR fluorescen* OR fluorescein* OR ICG OR ICGFA OR “Coloring Agents”[Mesh] OR “Coloring Agents”[Pharmacological Action] OR colouring OR coloring OR dye* OR “Fluorescent Dyes”[Pharmacological Action] OR "Fluorescein Angiography"[Mesh]) AND ("Anastomosis, Surgical"[Mesh] OR "Anastomotic Leak"[Mesh] OR anastomo* OR leak* OR "Perfusion"[Mesh] OR perfus* OR “blood supply”[Subheading] OR blood-suppl*))

Embase

('gastric surgery'/exp OR 'gastrectomy'/exp OR 'esophageal surgery'/exp OR 'esophagogastrectomy'/exp OR gastric resect* OR 'esophagect*' OR (('stomach'/exp OR 'gastric cancer'/exp OR 'esophagus'/exp OR 'esophageal cancer'/exp OR esophago-gastric OR early gastric cancer*) AND ('surgery':lnk OR surger* OR surgeo* OR surgi* OR resect* OR 'laparoscopy'/exp OR laparosc* OR laparoendosc* OR celioscop* OR 'minimally invasive surgery'/exp OR 'robotic surgical procedure'/exp))) AND ('indocyanine green'/exp OR 'fluorescence'/exp OR indocyan* OR 'indo cyan*' OR fluorescen* OR fluorescein* OR icg OR icgfa OR 'coloring agent'/exp OR colouring OR coloring OR dye* OR 'fluorescent dye'/exp) AND ('angiography'/exp OR 'perfusion assessment'/exp OR ‘vascularization’/exp OR ‘angiogr’/exp OR perfusion* OR angiography* OR blood supply'/exp OR blood suppl* OR ‘anastomotic leak’/exp OR anastomo*)
