# Supplementary figures and images for: European Association for Endoscopic Surgery (EAES) consensus on Indocyanine Green (ICG) fluorescence-guided surgery
Source: Surg Endosc. 2023 Feb 13;37(3):1629–48. doi: 10.1007/s00464-023-09928-5 (PMC10017637; doi:10.1007/s00464-023-09928-5)

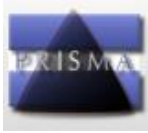

## PRISMA 2009 Flow Diagram – PERFUSION ASSESSMENT IN COLORECTAL SURGERY Setting

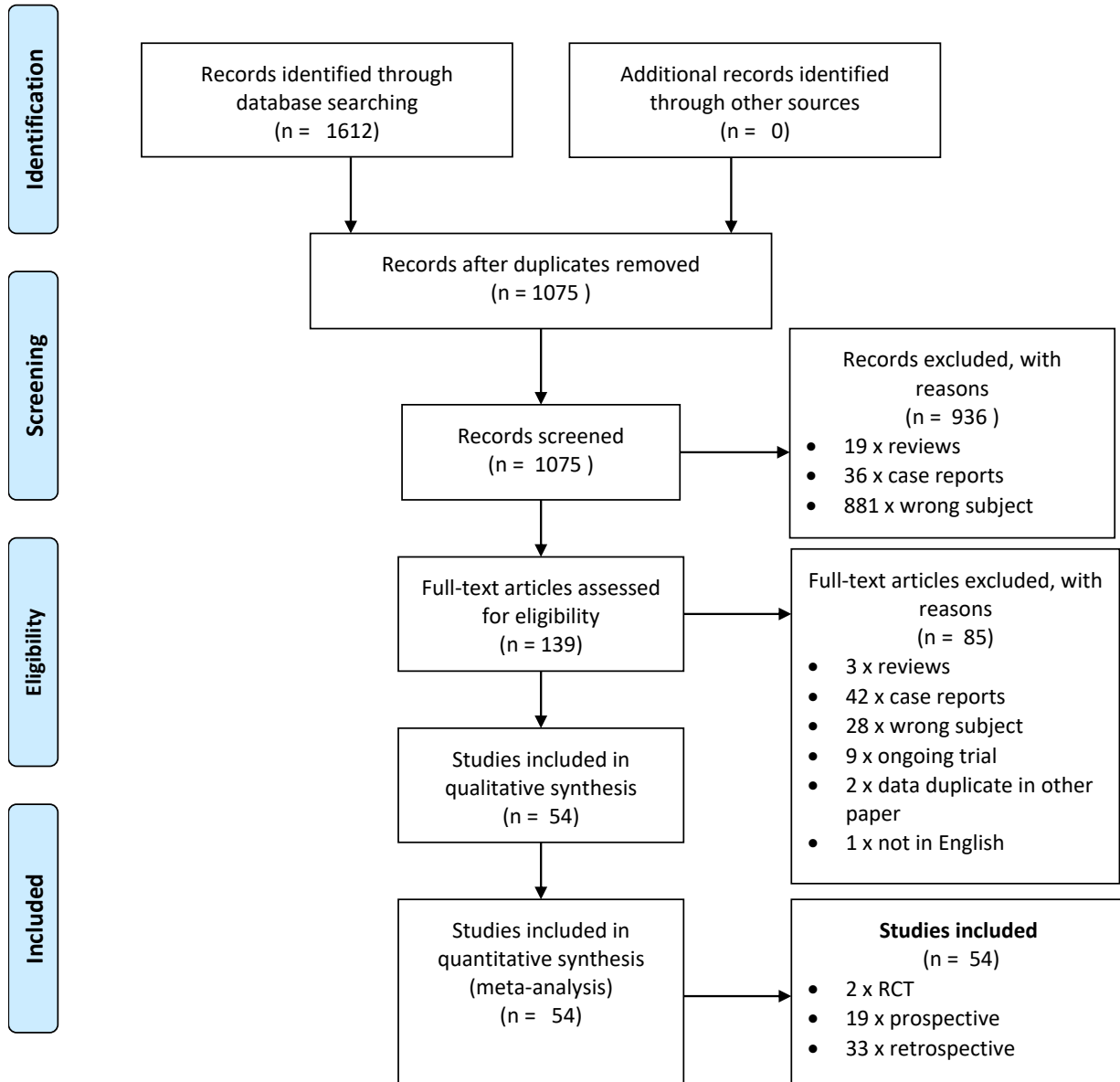

Supplement: Supplementary file 17 — Supplementary file17 (PDF 122 KB) [file 464_2023_9928_MOESM17_ESM.pdf]

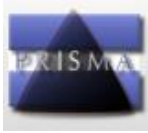

## PRISMA 2009 Flow Diagram – SPLEEN AND ADRENAL SURGERY Setting

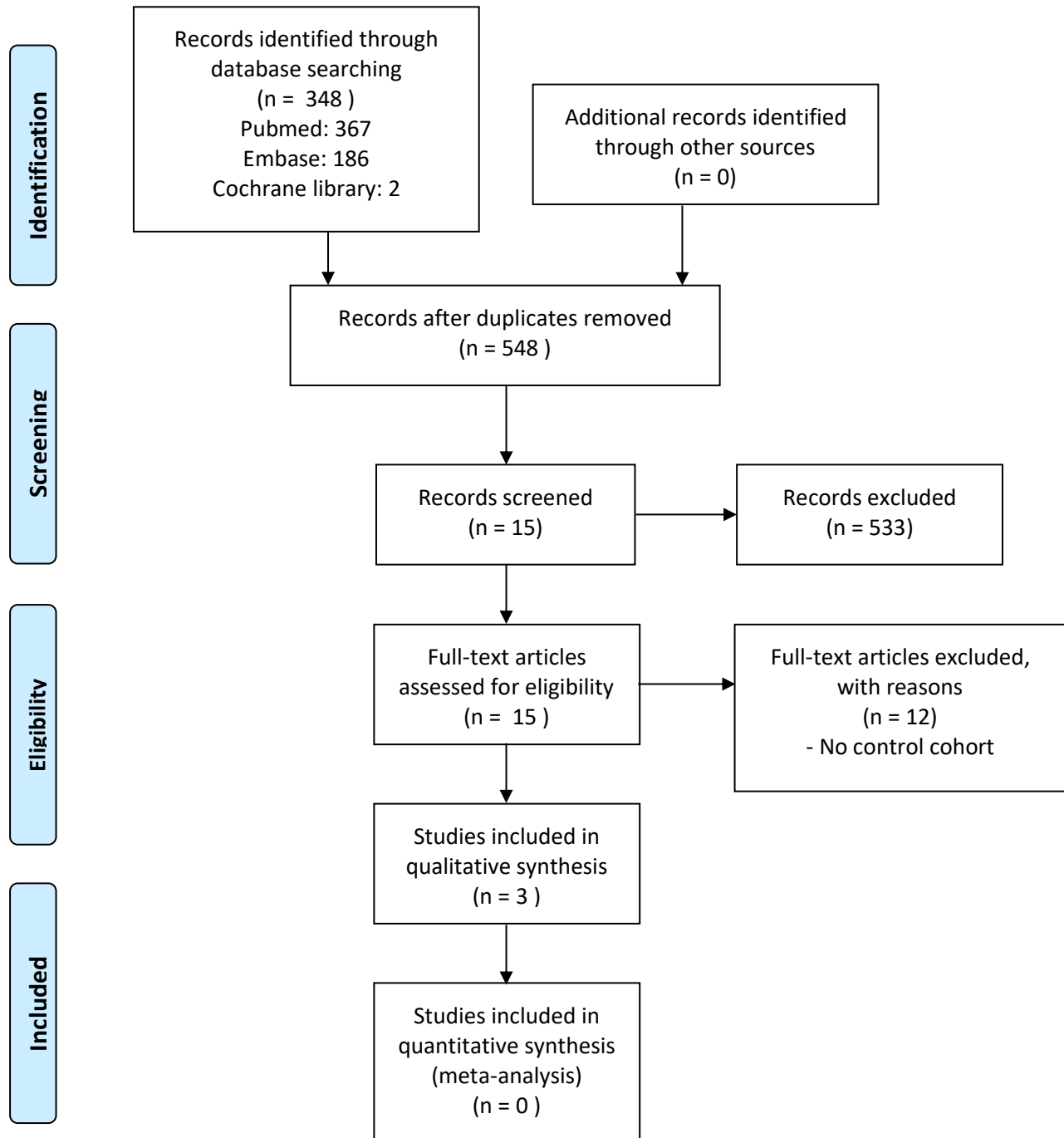

Supplement: Supplementary file 19 — Supplementary file19 (PDF 116 KB) [file 464_2023_9928_MOESM19_ESM.pdf]

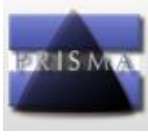

## PRISMA 2009 Flow Diagram

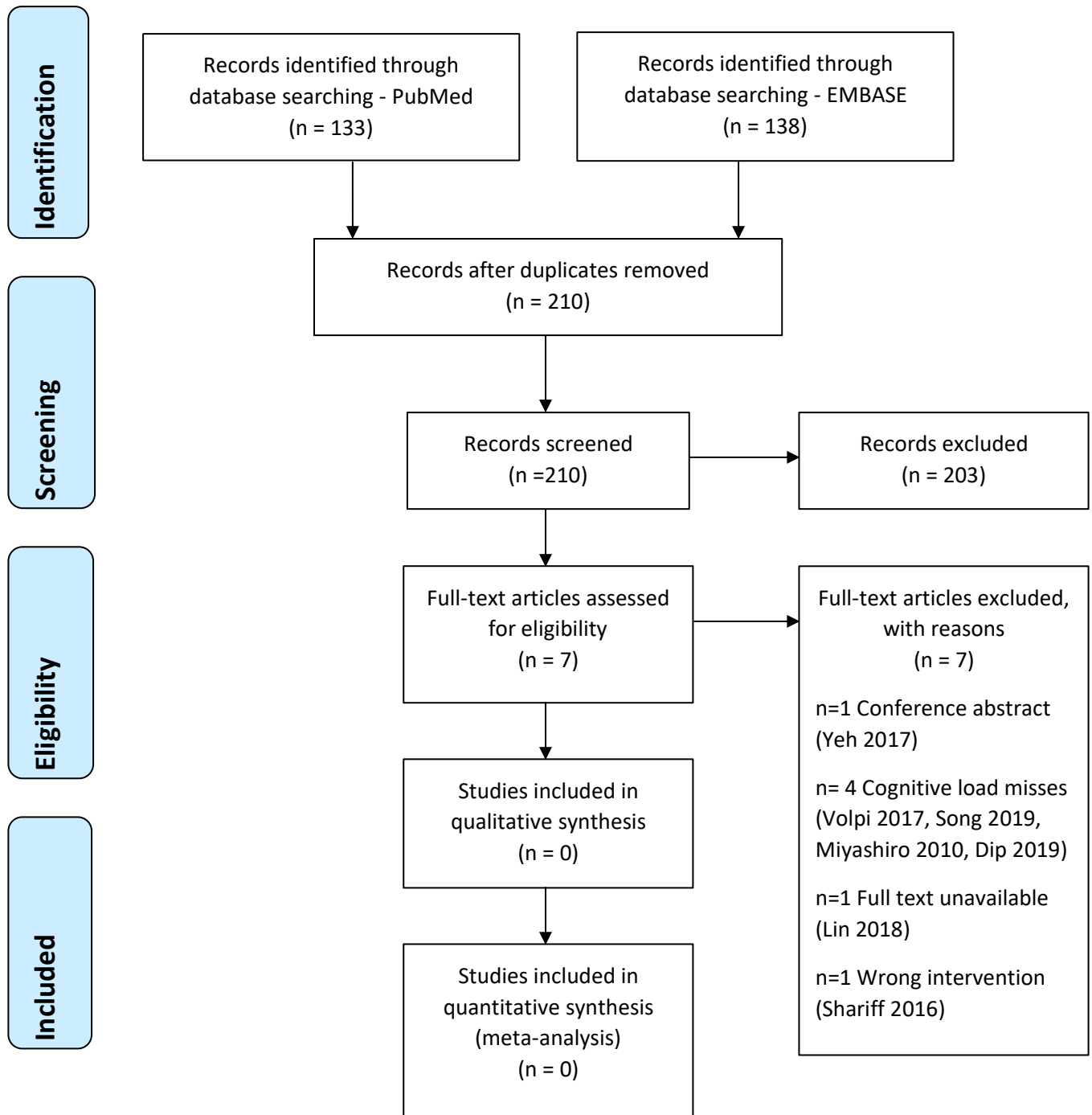

Supplement: Supplementary file 24 — Supplementary file24 (PDF 147 KB) [file 464_2023_9928_MOESM24_ESM.pdf]
