# Supplementary material for: European Association for Endoscopic Surgery (EAES) consensus on Indocyanine Green (ICG) fluorescence-guided surgery
Source: Surg Endosc. 2023 Feb 13;37(3):1629–48. doi: 10.1007/s00464-023-09928-5 (PMC10017637; doi:10.1007/s00464-023-09928-5)
Supplement: Supplementary file 20 — Supplementary file20 (PDF 130 KB) [file 464_2023_9928_MOESM20_ESM.pdf]

Surgery guided by indocyanine green enhanced fluorescence

Clinical question, PICOS and Search Strategy
Setting: Spleen and Adrenal Surgery

Clinical question: **Would indocyanine green - enhanced fluorescence surgery, rather than surgery without fluorescence - improve the outcome of patients after spleen and adrenal surgery?**

P = Population or Patient group: patients who underwent standard, laparoscopic or robotic surgery for adrenalectomy, partial/bilateral adrenalectomy, splenectomy.

I= Intervention:

- adrenalectomy with the use of ICG fluorescence for adrenal gland identification;
- adrenal sparing surgery with the use of ICG fluorescence for adrenal tumor identification
- splenectomy/partial splenectomy/splenopancreasectomy/spleen preserving surgery with ICG enhanced fluorescence

C= Comparator:

- adrenalectomy without the use of ICG fluorescence for adrenal gland identification;
- adrenal sparing surgery without the use of ICG fluorescence for adrenal tumor identification
- splenectomy/partial splenectomy/splenopancreasectomy/spleen preserving surgery without ICG enhanced fluorescence

O = Outcomes: organ and tumor identification, vascularization/ischemia assessment, intraoperative complications, operating time, mortality, morbidity

S = Study design

- Primary research: randomised controlled trials (RCTs), controlled cohort studies, case control studies
- Secondary research: systematic reviews and meta analysis

**Keyword A**	Indocyanine green - ICG				
**Keyword B**	near infreared fluorescence, fluorescence enhanced surgery				
**Keyword C**	Adrenalectomy, partial adrenalectomy, adrenal cancer				
**Keyword C**	Splenectomy, spleen preserving surgery				
**Search strategy**	Indocyanine green (ICG)	**OR**	near infreared fluorescence	**OR**	fluorescence enhanced surgery
**AND**	Adrenalectomy, partial adrenalectomy, adrenal cancer	**OR**	Splenectomy, spleen preserving surgery	**OR**	Endocrine surgery

Search methods for identification of studies: all sources searched, including: databases, trials registers, websites and grey literature; all types of studies included: case series, clinical trials, review and meta-analysys - **English language only**

Search Strategy

Pubmed

((((("Endocrine Surgery") OR "Endocrine Surgical Procedures"[Mesh])) OR (("adrenalectomy" OR "adrenal cancer" OR " splenectomy")))) AND (((((((("Indocyanine Green"[Mesh] OR "Fluorescent Dyes"[Mesh] OR “indocyanine green” OR wofaverdin OR vophaverdin OR fluorescen* OR cw800*)) OR ("near infrared fluorescence" OR "near infrared fluoresce imaging")))))))

Embase

((((((((endocrine surgery) OR splenectomy) OR adrenalectomy) OR "Endocrine Gland Neoplasms/surgery"[Mesh])) AND (((((((indocyanine green) OR fluorescent dyes) OR wofaverdin) OR vophaverdin) OR (fluorescence OR fluorescent)) OR cw800*) OR "near infrared"))) AND ((indocyanine) OR Indocyanine Green OR wofaverdin OR vophaverdin)
