# Supplementary material for: European Association for Endoscopic Surgery (EAES) consensus on Indocyanine Green (ICG) fluorescence-guided surgery
Source: Surg Endosc. 2023 Feb 13;37(3):1629–48. doi: 10.1007/s00464-023-09928-5 (PMC10017637; doi:10.1007/s00464-023-09928-5)
Supplement: Supplementary file 21 — Supplementary file21 (PDF 99 KB) [file 464_2023_9928_MOESM21_ESM.pdf]

Surgery guided by indocyanine green enhanced fluorescence

Clinical question, PICOS and Search Strategy

Setting: prostatectomy for prostate cancer

Clinical question: **Would indocyanine green - enhanced fluorescence surgery, rather than surgery without fluorescence - improve the outcome of patients after prostatectomy?**

P = Population or Patient group: patients who underwent standard, laparoscopic or robotic assited radical prostatectomy for prostate cancer
I= Intervention: surgical procedure (standard, laparoscopic, robotic) with fluorescent properties of indocyanine green (ICG)
C= Comparator: surgical procedure (standard, laparoscopic, robotic) without fluorescent properties of indocyanine green (ICG)
O = Outcomes: mortality, morbidity**,** operating time, re-operation, re-admission
S = Study design

- Primary research: <u>randomised controlled trials (RCTs), controlled cohort studies, case control studies</u>
- Secondary research: systematic reviews and meta analysis

**Keyword A**	**Prostatectomy**				
**Keyword B**	**Indocyanine Green**				
**Keyword C**	**near infrared fluorescence**				
**Keyword C**					
**Search strategy**	**Prostatectomy**	**AND**	**Indocyanine Green**	**AND**	**near infrared fluorescence**
AND	**Fluorescent Dyes[Mesh]**	**AND**	**vophaverdin**		

<u>Search methods for identification of studies:</u>

all sources searched, including: databases, trials registers, websites and grey literature; all types of studies included: case series, clinical trials, review and meta-analysys
English language only

Electronic sources of published literature: Pubmed, Embase, Cochrane Library

(((((prostatectomy) OR "Prostatectomy"[Mesh])) AND ((((("Indocyanine Green"[Mesh] OR "Fluorescent Dyes"[Mesh] OR “indocyanine green” OR wofaverdin OR vophaverdin OR fluorescen* OR cw800*)) OR ("near infrared fluorescence" OR "near infrared fluoresce imaging"))))

Surgery guided by indocyanine green enhanced fluorescence

Clinical question, PICOS and Search Strategy

Setting: Radical Cystectomy for bladder cancer

Clinical question: **Would indocyanine green - enhanced fluorescence surgery, rather than surgery without fluorescence - improve the outcome of patients after radical cystectomy?**

P = Population or Patient group: patients who underwent standard, laparoscopic or robotic assited radical cystectomy for muscle invasive bladder cancer
I= Intervention: surgical procedure (standard, laparoscopic, robotic) with fluorescent properties of indocyanine green (ICG)
C= Comparator: surgical procedure (standard, laparoscopic, robotic) without fluorescent properties of indocyanine green (ICG)
O = Outcomes: mortality, morbidity**,** operating time, re-operation, re-admission
S = Study design

- Primary research: randomised controlled trials (RCTs), controlled cohort studies, case control studies
- Secondary research: systematic reviews and meta analysis

**Keyword A**	**Cystectomy**				
**Keyword B**	**Indocyanine Green**				
**Keyword C**	**near infrared fluorescence**				
**Keyword**					
**Search strategy**	**Cystectomy**	**AND**	**Indocyanine Green**	**AND**	**near infrared fluorescence**
AND	**Fluorescent Dyes[Mesh]**	**AND**	**vophaverdin**		

Search methods for identification of studies:

all sources searched, including: databases, trials registers, websites and grey literature; all types of studies included: case series, clinical trials, review and meta-analysys
English language only

Electronic sources of published literature: Pubmed, Embase, Cochrane Library

((("Cystectomy") OR "Cystectomy"[Mesh])) AND ((((("Indocyanine Green"[Mesh] OR "Fluorescent Dyes"[Mesh] OR “indocyanine green” OR wofaverdin OR vophaverdin OR fluorescen* OR cw800*)) OR ("near infrared fluorescence" OR "near infrared fluoresce imaging"))))

Surgery guided by indocyanine green enhanced fluorescence

Clinical question, PICOS and Search Strategy

Setting: Radical or Partial Nephrectomy for kidney cancer

Clinical question: **Would indocyanine green - enhanced fluorescence surgery, rather than surgery without fluorescence - improve the outcome of patients after nephrectomy?**

P = Population or Patient group: patients who underwent standard, laparoscopic or robotic assited radical/partial nephrectomy for kidney cancer
I= Intervention: surgical procedure (standard, laparoscopic, robotic) with fluorescent properties of indocyanine green (ICG)
C= Comparator: surgical procedure (standard, laparoscopic, robotic) without fluorescent properties of indocyanine green (ICG)
O = Outcomes: mortality, morbidity**,** operating time, re-operation, re-admission
S = Study design

- Primary research: randomised controlled trials (RCTs), controlled cohort studies, case control studies
- Secondary research: systematic reviews and meta analysis

**Keyword A**	**Nephrectomy**				
**Keyword B**	**Indocyanine Green**				
**Keyword C**	**near infrared fluorescence**				
**Keyword**					
**Search strategy**	**Nephrectomy**	**AND**	**Indocyanine Green**	**AND**	**near infrared fluorescence**
AND	**Fluorescent Dyes[Mesh]**	**AND**	**vophaverdin**		

Search methods for identification of studies:

all sources searched, including: databases, trials registers, websites and grey literature; all types of studies included: case series, clinical trials, review and meta-analysys
English language only

Electronic sources of published literature: Pubmed, Embase, Cochrane Library

Search Strategy

((("Nephrectomy") OR "Nephrectomy"[Mesh])) AND ((((("Indocyanine Green"[Mesh] OR "Fluorescent Dyes"[Mesh] OR "indocyanine green" OR wofaverdin OR vophaverdin OR fluorescen* OR cw800*)) OR ("near infrared fluorescence" OR "near infrared fluoresce imaging"))))
