# Supplementary material for: European Association for Endoscopic Surgery (EAES) consensus on Indocyanine Green (ICG) fluorescence-guided surgery
Source: Surg Endosc. 2023 Feb 13;37(3):1629–48. doi: 10.1007/s00464-023-09928-5 (PMC10017637; doi:10.1007/s00464-023-09928-5)
Supplement: Supplementary file 23 — Supplementary file23 (PDF 84 KB) [file 464_2023_9928_MOESM23_ESM.pdf]

Searchstrings – cognitive load dd 02-12-2019

PubMed:

("Imaging, Fluorescence"[Mesh] OR “Fluorescence guided surgery”[Mesh] OR Fluorescence* OR ICG* OR fluorescence-guided-surgery* OR fluorescence*[title])
AND ("Laparoscopy"[Mesh] OR "Laparoscopes"[Mesh] OR laparosc* OR laparoendosc* OR celioscop* OR "Minimally Invasive Surgical Procedures"[Mesh:NoExp] OR minimally-invasive-surg*) AND (“Cognition”[Mesh] OR “Learning”[Mesh] OR “Task performance and Analysis”[Mesh] OR cognitive-load* OR workload* OR working-load* OR work-load* OR task* OR learn* OR memor* OR effort* OR instruction* OR skill*[tiab] OR competenc*[tiab] OR proficien*[tiab] OR performance*[tiab] OR cognitive*[tiab] or education*)

EMBASE”:

'fluorescence surgery imaging'/exp or 'fluorescence' or 'fluorescence-guided-surgery*'OR 'fluorescence-surgery*' OR 'fluorescence guided surgery' OR 'fluorescence surgery' AND 'laparoscopy'/exp OR 'laparoscope'/exp OR laparosc* OR laparoendosc* OR celioscop* OR 'minimally invasive surgery'/de OR 'minimally invasive surg*') AND ('cognition'/exp OR 'learning'/exp OR 'task performance'/exp OR cognitive-load* OR workload* OR working-load* OR work-load* OR task* OR learn* OR memor* OR effort* OR instruction* OR skill*:ti,ab OR competenc*:ti,ab OR proficien*:ti,ab OR performance*:ti,ab
